# Rapid propagation of membrane tension at retinal bipolar neuron presynaptic terminals

**DOI:** 10.1126/sciadv.abl4411

**Published:** 2022-01-05

**Authors:** Carolina Gomis Perez, Natasha R. Dudzinski, Mason Rouches, Ane Landajuela, Benjamin Machta, David Zenisek, Erdem Karatekin

**Affiliations:** 1 Department of Cellular and Molecular Physiology, Yale University, New Haven, CT, USA.; 2 Nanobiology Institute, Yale University, West Haven, CT, USA.; 3 Interdepartmental Neuroscience Program, Yale University, New Haven, CT, USA.; 4 Department of Molecular Biophysics and Biochemistry, Yale University, New Haven, CT, USA.; 5 Systems Biology Institute, Yale University, West Haven, CT, USA.; 6 Department of Physics, Yale University, New Haven, CT, USA.; 7 Kavli Institute for Neuroscience, Yale University, New Haven, CT, USA.; 8 Department of Neuroscience, Yale University, New Haven, CT, USA.; 9 Department of Ophthalmology and Visual Sciences, Yale University, New Haven, CT, USA.; 10 Université de Paris, SPPIN—Saints-Pères Paris Institute for the Neurosciences, Centre National de la Recherche Scientifique (CNRS), Paris F-75006, France.

## Abstract

Many cellular activities, such as cell migration, cell division, phagocytosis, and exo-endocytosis, generate and are regulated by membrane tension gradients. Membrane tension gradients drive membrane flows, but there is controversy over how rapidly plasma membrane flow can relax tension gradients. Here, we show that membrane tension can propagate rapidly or slowly, spanning orders of magnitude in speed, depending on the cell type. In a neuronal terminal specialized for rapid synaptic vesicle turnover, membrane tension equilibrates within seconds. By contrast, membrane tension does not propagate in neuroendocrine adrenal chromaffin cells secreting catecholamines. Stimulation of exocytosis causes a rapid, global decrease in the synaptic terminal membrane tension, which recovers slowly due to endocytosis. Thus, membrane flow and tension equilibration may be adapted to distinct membrane recycling requirements.

## Teaser

Cell membranes flow very differently across cell types, likely reflecting distinct membrane recycling activities.

## INTRODUCTION

**Fig. 1.**
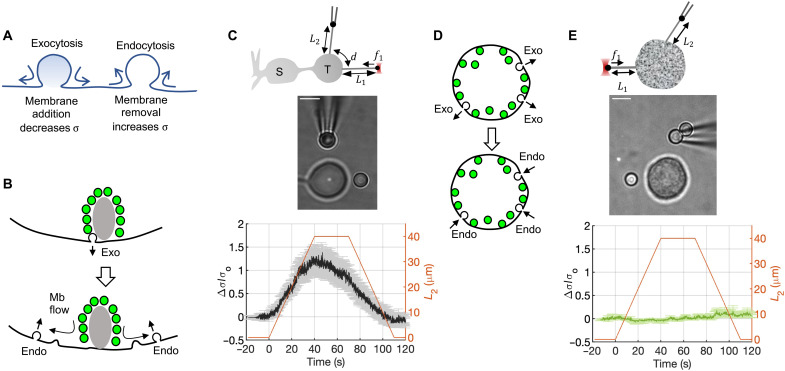
Membrane tension propagates rapidly in neuronal synaptic terminals, but not in endocrine chromaffin cells. (**A**) Exocytosis adds area to the plasma membrane, locally decreasing membrane tension, σ. Endocytosis locally increases σ through removal of plasma membrane. Exo- and endocytosis occurring at distinct loci imply net membrane transfer from exocytic-to-endocytic sites. (**B**) In retinal bipolar terminals, release predominantly occurs at active zones marked by ribbons (gray oval). Most endocytosis occurs at other sites, distributed throughout the terminal. Mb, membrane. (**C**) Testing membrane flow at bipolar cell terminals. Top: Schematic of the experiment. A “probe” tether is pulled from a terminal using a bead held in an OT. A second “pulling” tether is extended from the terminal using another bead held by a micropipette. While the length *L*_2_ of the pulling tether is varied, the probe tether is held stationary and its tension is measured through the force acting on the bead held in the OT. S, soma; T, terminal. Middle: Snapshot from an experiment. Only the terminal and part of the axon are visible. Bottom: Change in membrane tension ∆σ of the probe tether relative to its resting value, σ_0_, as a function of time (left axis, *n* = 12 cells). Error bars (gray) indicate standard error. Intertether distance was *d* = 4 to 11 μm. *L*_2_ is shown on the right axis (red). (**D**) In adrenal chromaffin cells, most secretory granules undergoing exocytosis are retrieved at the same loci. (**E**) Same as in (C) but for neuroendocrine chromaffin cells (*n* = 6, *d* = 6 to 12 μm). Scale bars, 5 μm. The medians of the maximal values of Δσ/σ_0_ are different for bipolar cell terminals and chromaffin cells (*P* < 0.01, Wilcoxon rank sum test).

Neuronal presynaptic terminals are hubs of intense and rapid membrane turnover. Upon stimulation, the equivalent area of the synaptic vesicles (SVs) that fuse rapidly with the presynaptic plasma membrane is recovered through compensatory endocytosis to restore cell membrane area and to maintain a releasable pool of SVs (Fig. 1A). How exocytosis triggers endocytosis is under debate (*1*, *2*); a possible mechanism involves sensing a drop in membrane tension at the endocytic site caused by exocytic membrane addition some distance away (*3*), supported by the observation that even modest increases in membrane tension strongly inhibit endocytosis (*4*–*6*). However, recent work indicated that membrane tension gradients in several cell types do not equilibrate over micrometer length scales for tens of minutes, implying extremely slow membrane flows (*7*). Membrane tension propagating this slowly could not possibly act as a signal to trigger rapid compensatory endocytosis even at sites a few hundred nanometers away from typical release sites (*3*). Even if a signal other than a decrease in membrane tension triggered endocytosis, membrane flow would be too slow to supply membrane to endocytic locations in the periphery of active zones, at least in terminals with rapid SV turnover (*8*). These results suggest either that synaptic terminals must be specialized for facile membrane flow or that sensing exocytosis and supplying membrane to endocytic sites must occur via other means.

Here, we used goldfish retinal bipolar neurons as a model to test these possibilities. They have a single giant (~10 μm) terminal, filled with up to 50 active zones marked by a ribbon, a dense structure to which approximately 100 SVs are attached and at the base of which most release takes place (*9*, *10*). Each terminal contains a total pool of ~0.5 million SVs (*11*, *12*). These cells release glutamate rapidly [time constants (*13*) of ~1 to 2 ms and 150 ms] to graded potentials: up to ~10 to 15% of the initial area can be added to the terminal within 250 ms upon strong stimulation (*13*–*16*). Endocytosis restores terminal membrane area at longer time scales [~2 and ~30 s for weak and strong stimuli (*14*)]. In these cells, most endocytosis is known to occur away from ribbon sites where exocytosis occurs (*17*, *18*), implying relatively fast membrane flow (Fig. 1B). As a comparison, we tested membrane tension propagation and flows in neuroendocrine adrenal chromaffin cells. In contrast to neurons, these cells release their cargo in less spatially restricted regions into the bloodstream, undergoing exocytosis and endocytosis in overlapping regions (*19*, *20*), which does not necessitate long-range membrane flows (Fig. 1D and fig. S1).

## RESULTS

### Membrane tension dynamics are very different in retinal bipolar neurons and adrenal chromaffin cells

To probe propagation of membrane tension in bipolar cell presynaptic terminals, we pulled a pair of thin membrane tethers from the cell surface using 3-μm latex beads as handles (Fig. 1C). First, a short “probe tether” was pulled using an optical trap (OT) and maintained at constant length. Then, a second, “pulling tether” was extruded a few micrometers using a bead manipulated by a micropipette mounted on a programmable piezo stage. After equilibration, we extended the pulling tether at 1μm/s for 40 μm, held it for 30 s, and then relaxed it to its initial position while monitoring tension changes in the probe tether using OT. Extension of the pulling tether caused a local increase in cell membrane tension (fig. S2). Propagation of this local change to the probe tether was monitored via the force acting on the probe tether using OT, $f_{t}=2\pi \sqrt{2\kappa \sigma_{t}}$, where σ*_t_* is the tether membrane tension and κ ≈ 0.27 pN∙μm is the bending modulus (*21*). The tether force is deduced from deviations of the bead’s position from the center of the OT and known calibration of the trap stiffness, allowing us to estimate σ*_t_* (Materials and Methods). To prevent spontaneous depolarizations, which lead to exo-endocytosis (*22*) that could perturb membrane tension measurements, we maintained cells in a low extracellular calcium solution.

Membrane tension perturbations created by the pulling tether were transmitted rapidly, within at most a few seconds, to the probe tether, despite being separated by 4 to 11 μm (Fig. 1C and movie S1). As the pulling tether was extended, increasing tension in the tether and its base (fig. S2), tension in the probe tether increased. When extension of the pulling tether stopped, tension in the probe tether started relaxing. When the length of the pulling tether was decreased, tension in the probe tether decreased (Fig. 1C).

Additional experiments confirmed these observations and ruled out potential artifacts. First, using similar double-tether experiments, we confirmed that in HeLa cells membrane tension does not propagate over micrometer length scales within several minutes (fig. S3), consistent with a recent report (*7*). Second, we confirmed that tethers pulled from bipolar cells are cytoskeleton-free (fig. S4), as the force-tension relationship above assumes. Third, we confirmed that membrane tension in the probe tether tracked tension changes in the pulling tether by monitoring fluorescence changes simultaneously in the two tethers (fig. S5). Last, to test whether the results depended on how rapidly we moved the pulling tether, we repeated the measurements using a twofold slower speed, qualitatively obtaining the same result (fig. S6). We conclude that membrane tension equilibrates over several micrometers within a few seconds in presynaptic terminals, in stark contrast to other cell types (*7*).

To probe propagation of membrane tension in the soma, we repeated the double-tether experiments of Fig. 1C but pulled both tethers from the soma, with intertether distances of 4 to 12 μm. We found the membrane tension perturbation created by the pulling tether was again transmitted rapidly to the probe tether, but the change in membrane tension relative to its initial value was smaller at the probe tether (fig. S7). These results were consistent with fluorescence-based simultaneous tension estimates of the two tethers (fig. S5E). The resting tensions covered a broad range and were indistinguishable between somas and terminals, while the tension perturbation created by extending a tether increased in a similar manner (fig. S2, C to E). Thus, membrane tension also propagates rapidly in the soma. Terminal-to-axon transmission of membrane tension changes was also rapid, with a slightly lower amplitude, but intertether distances were larger (14 to 17 μm; fig. S7).

As a comparison, we tested how rapidly tension propagates in neuroendocrine chromaffin cells, where endo- and exocytosis instead occur in overlapping regions, using double-tether experiments as above. Extending the pulling tether strongly increased membrane tension locally (fig. S2), yet we could not detect membrane tension propagation over 6 to 12 μm in several minutes in any of the six cells tested (Fig. 1E and movie S2).

We conclude that membrane tension propagates very rapidly in bipolar neurons and unmeasurably slowly in adrenal chromaffin cells, two cell types specialized for secretion but with different spatiotemporal secretory vesicle dynamics, the latter not requiring long-range plasma membrane transport.

### Neither tracer diffusion nor obstacle density correlates with membrane tension propagation

We tested whether a difference in the mobility of integral transmembrane domain (TMD) proteins could explain the large differences in membrane flows we observed among chromaffin cells, bipolar neuronal terminals, and somas. We labeled surface proteins with an organic dye and used fluorescence recovery after photobleaching (FRAP), fitting measured curves to find an immobile fraction and a diffusion constant of mobile labeled proteins (fig. S8). Unexpectedly, we found no substantial difference among the tracer diffusivities for the three cases [*D_t_* = (10 ± 6.7) × 10^−3^, (19 ± 5.8) × 10^−3^, and (11 ± 4.8) × 10^−3^ μm^2^/s for chromaffin cells, terminals, and somas, respectively]. The immobile fraction was similar for the terminal (0.39 ± 0.02) and soma (0.37 ± 0.04), which were ~30% lower than for chromaffin cells (0.60 ± 0.03).

**Fig. 2.**
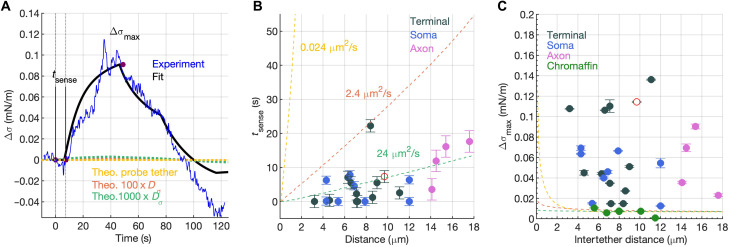
Rapid membrane tension propagation in bipolar cell terminals is unlikely to be diffusive. (**A**) Comparison of measured and predicted membrane tension changes ∆σ at the probe tether as the pulling tether creates a controlled tension perturbation a distance *d* away (see Fig. 1). A model predicting diffusive propagation of σ (*7*) was fitted to experimentally measured ∆σ. In this example, *d* = 9.7 μm. Membrane tension diffusion coefficients varying from the value estimated (*7*) for HeLa cells (*D*_σ_ = 0.024 μm^2^/s) to a value 10^3^-fold larger did not produce a good fit. Good agreement was obtained only when *d* = 0.1 μm was assumed (black), suggesting rapid propagation of tension with weak distance dependence. (**B**) Time to sense at the probe tether a tension change at the pulling tether, *t*_sense_, as a function of *d*. To estimate *t*_sense_, we used the difference between the actual time the pulling tether was set in motion (*t* = 0) and the onset of the predicted best-fit tension change at the pulling tether [black curve in (A)]. Filled circles are experimental measurements. The red empty circle corresponds to the trace in (A). The values of *t*_sense_ predicted by diffusive propagation of σ are indicated as dashed curves, with the corresponding values of *D*_σ_. The region above the yellow curve corresponds to values of *t*_sense_ allowed by the model and measurements in (*7*). (**C**) Maximum change in Δσ at the probe tether as a function of *d*. The amplitude-distance relationship predicted by the diffusive propagation model is indicated by the dashed curves [same color scheme as in (A) and (B)]. Filled circles are the experimental values, and the empty red circle corresponds to the trace in (A) (see the Supplementary Materials and fig. S9).

We wanted to understand whether the differences in TMD protein immobile fractions could explain the differences in membrane tension propagation. The presence of immobile obstacles can markedly hinder bulk flow even while tracer diffusion remains relatively unimpeded, especially in two dimensions (2D) (*7*, *23*). We tested whether a recent model (*7*), where TMD proteins that interact with the underlying cytoskeleton represent a random array of fixed obstacles to flow, could explain our observations. The model predicts diffusive membrane tension propagation, with a tension diffusion coefficient *D*_σ_ = *Ek*/η, where *E* is the membrane stretch modulus, η is the 2D membrane viscosity, and *k* is the Darcy permeability of the array of obstacles. In a model where obstacles are randomly arranged in space, the Darcy permeability is a function of the obstacle area fraction ϕ and the radius *a* of obstacles (*7*, *23*), *k* = *a*^2^*f*(ϕ), where *f*(ϕ) is a rapidly decaying function of ϕ. We estimated ϕ from the immobile membrane protein fraction, assuming that ~25% of membrane area is occupied by transmembrane proteins (*24*, *25*), ϕ ≈ 0.093 ± 0.01 and ϕ ≈ 0.096 ± 0.005 in the soma and terminal, respectively, and ϕ ≈ 0.150 ± 0.008 in chromaffin cells, yielding *k* ≈ 7.45 ± 1.23 nm^2^ and 6.96 ± 0.59 nm^2^ for the soma and terminal, respectively, and *k* ≈ 3.01 ± 0.30 nm^2^ for chromaffin cells. Using similar values for *a*, η, and *E* for bipolar and chromaffin cells, only a 2.4-fold difference in the tension diffusion coefficient *D*_σ_ would be expected. This is insufficient to explain the differences of membrane tension propagation between chromaffin cells and bipolar neurons. Using numerical calculations, we estimate that 100- to 1000-fold larger *D*_σ_ is needed to explain the rapid membrane flow we observe in bipolar cells if tension propagates diffusively (Fig. 2A; see also the Supplementary Materials and fig. S9). In addition, the speed and amplitude of tension propagation show weak distance dependence (Fig. 2, B and C), suggesting that the underlying propagation mechanism may not be diffusive in bipolar neurons.

In summary, neither tracer diffusion nor the fraction of immobile obstacles can explain the large differences in membrane tension propagation we observe in bipolar and chromaffin cells. Thus, while our results cannot rule out a model in which the cell membrane flows are heavily disturbed by immobile TMD proteins (*7*), other factors likely contribute to the propagation of membrane tension in bipolar cells.

### Plasma membrane–cytoskeleton drag correlates with membrane tension propagation

The dominant resistance to membrane flow in cell membranes arises from interactions between the plasma membrane and the adjacent layer of the underlying cytoskeleton (*7*, *26*). Forcing membrane tethers to slide on the cell surface and monitoring the force-velocity relationship provides a measure of this resistance (*27*).

**Fig. 3.**
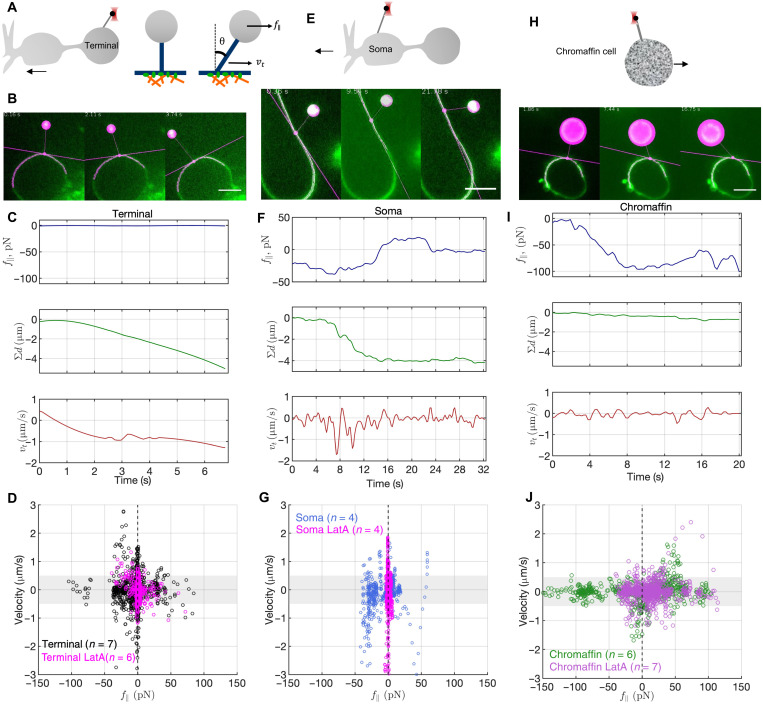
Membrane tethers extruded from bipolar neurons, but not from chromaffin cells, can be dragged with ease. (**A**) Schematic of the experiment. After a membrane tether is extruded from a terminal, the cell is moved to generate a tangential component *f*_∥_ of the tether force, given by the sine of the tether membrane angle θ. The velocity *v_t_* with which the tether base moves is recorded. (**B**) Snapshots from a tether-dragging experiment at a bipolar cell terminal. The detected contour of the cell membrane is shown in magenta. The intersection of the tether with the cell surface is indicated by a magenta point, and the tangent at that point by a line. The bead is automatically detected and overlaid by a disc. Cell membranes were visualized with CellMask Deep Red or FM4-64. (**C**) Time profiles of the tangential force, *f*_∥_, cumulative distance traveled from origin, ∑*d*, and frame-to-frame tether velocity, *v_t_*. Scale bar, 5 μm. (**D**) Tether velocity–tangential force pairs for untreated (black) or LatA-treated (20 μM, 20 min, magenta) terminals. The gray area, ∣*v_t_* ∣ < 0.5 μm/s, corresponds to the level of noise in *v_t_* and may be considered stationary. (**E** to **G**) Similar measurements for tethers drawn from the somas of bipolar neurons. (**H** to **J**) Similar measurements for tethers drawn from adrenal chromaffin cells. For bipolar cell terminals, and to a lesser extent for somas, very small forces were sufficient to set a tether in motion, whereas tethers drawn from chromaffin cells required much larger forces to slide. LatA treatment reduced forces in all cases, although there was no visible blebbing under these conditions.

We found that most tethers (17 of 20) could be dragged with ease around the bipolar cell terminal as soon as a finite tangential force was generated by moving the cell with respect to the bead holding the end of the tether (Fig. 3, A and B, and movie S3). Occasionally, tethers were transiently immobilized for 1 to 3 s. Combining image analysis with force measurements, we quantified, *f*_∥_, the force tangent to the terminal membrane and the distance traveled by the tether base for every frame (Fig. 3C). From the distance profile, we computed the frame-to-frame velocities. Tether base velocity as a function of tangential force is shown in Fig. 3D (black circles). Low forces (~8 pN) were sufficient to produce large velocities. Tethers moved mostly unimpeded, interspersed with transient pauses, likely due to the heterogeneous nature of the underlying cytoskeleton. By contrast, most tethers extruded from chromaffin cells (six of eight) were immobile. Tethers that did slide here required much larger forces (~24 pN) and paused more frequently after initial sliding for ~0.25 to 1 μm (Fig. 3, H to J, and movie S4). Tethers dragged from bipolar cell somas displayed intermediate behavior (Fig. 3, E to G; fig. S10; and movie S5).

Disruption of the actin cortex using latrunculin A [LatA; preventing actin polymerization (*28*); fig. S11] facilitated tether sliding in terminals and somas and, to a lesser extent, in chromaffin cells (Fig. 3, D, G, and J, and fig. S10). After treatment with 20 μM LatA for 20 min, which does not lead to visible bleb formation, most tethers drawn from somas (six of eight) could be dragged with ease and supported forces (⟨*f*_∥_⟩ ≈ 1.6 pN) similar to those in treated terminals (~6 pN). Tethers from treated chromaffin cells slid readily (four of seven), albeit at high angles and with higher forces (~23 pN) than untreated termini or somas.

Together, these results suggest that the cytoskeleton impedes tether sliding to a much higher degree in chromaffin cells than in bipolar cell somas. Tethers drawn from terminals display minimal resistance to tether dragging, despite having a readily detectable F-actin cortex [(*29*, *30*) and figs. S4 and S11].

### Neuronal activity drives rapid changes in presynaptic membrane tension

Membrane area changes of the terminal have been monitored directly using time-resolved capacitance (*14*, *15*, *31*): The excess area added to the terminal upon mild stimulation (up to 0.2-s depolarization to 0 mV, leading to fusion of ~2000 SVs, or ~8% of the terminal area) is recovered through rapid endocytosis (time constant ~1 s), whereas membrane area increases elicited by stronger stimulation are restored over ~20 to 30 s. If SVs fuse completely with the active zone membrane (*17*, *18*) and tension perturbations propagate rapidly in the terminal, we expect to observe a rapid, terminal-wide decrease in membrane tension upon strong stimulation of exocytosis, followed by slower recovery due to endocytosis.

To test this hypothesis, we used two approaches. First, we took advantage of the fact that some bipolar cells spontaneously develop calcium action potentials that drive exocytosis (*22*). We loaded cells with a calcium indicator, Fluo-4-AM, to monitor calcium fluctuations (*32*) while simultaneously measuring membrane tension at the terminal using OT in the presence of extracellular calcium. Occasionally, cells displayed robust calcium activity. Calcium spikes anti-correlated with membrane tension (fig. S12), consistent with calcium-stimulated exocytosis reducing membrane tension and endocytosis driving its recovery (*22*).

**Fig. 4.**
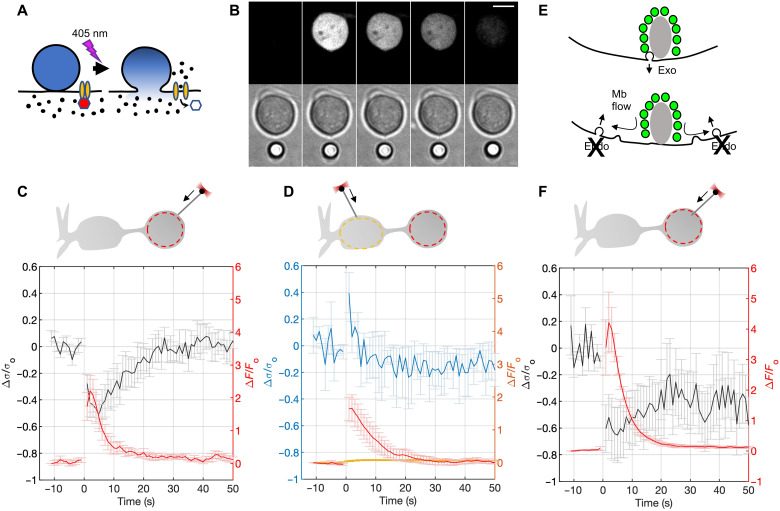
Activity-dependent changes in membrane tension in bipolar neurons. (**A**) Principle of photostimulation used. Nifedipine (depicted as a red hexagon) blocks L-type calcium channels. Photolysis at 405 nm removes the block, causing calcium entry and exocytosis (*36*). (**B**) Example of a simultaneous calcium imaging and tether force measurement during photostimulation of a bipolar cell terminal. A membrane tether was pulled from a bipolar cell terminal and membrane tension monitored, while nifedipine in the bath (20 μM) was photolyzed by a 1-s 405-nm pulse. The cell was preloaded with the calcium indicator Fluo-4. Top row: Fluo-4 fluorescence. Bottom row: Bright-field imaging. The two channels were alternated during acquisition. The tether becomes visible upon stimulation and calcium increase (second column), indicating a sharp decrease in tether tension. Scale bar, 5 μm. (**C**) Fractional changes in membrane tension (black, left axis) and calcium signals (red, right axis) averaged over six experiments. Photolysis was applied for 1 s starting at *t* = 0 s. Membrane tension recovered completely within 50 s after stimulation. The ROI used for fluorescence quantification is indicated in the schematic as a dashed circle. (**D**) Similar to (C), but the tether forces were measured in the soma (*n* = 4). Most L-type calcium channels are in the terminal where calcium dynamics are largely confined (*34*). (**E**) Inhibition of endocytosis by MiTMAB, a dynamin inhibitor (*37*, *38*), is expected to reduce recovery of membrane tension following exocytosis. (**F**) Similar to (C), but measurements were made in the presence of 30 μM MiTMAB to block endocytosis (*n* = 4). Only ~30% of the initial drop in membrane tension upon stimulation recovered. See fig. S12 for additional experiments.

Second, to exert better control over the release process, we used a photochemical approach to stimulate exo- and endocytosis (Fig. 4A). Neurotransmitter release in type Mb bipolar cells is driven by calcium entry via L-type calcium channels (*33*, *34*). To stimulate release, we bathed the cells in nifedipine (20 μM), a blocker of L-type dihydropyridine (DHP)-sensitive calcium channels (*35*). Photolysis of nifedipine at 405 nm removes the block, allowing calcium influx and exocytosis (*36*). We loaded cells with a calcium indicator, Fluo-4-AM, to monitor calcium entry upon stimulation (*32*). We pulled a membrane tether and monitored its tension while imaging calcium signals using time-lapse spinning-disc confocal microscopy. Stimulation with 405-nm illumination for 1 s caused a rapid increase in the calcium signal (Fig. 4, B and C). There was a concomitant decrease in membrane tension with a characteristic time of ~1 to 2 s. Although the cytosolic calcium increase and the accompanying exocytosis occur instantly at the time resolution of these recordings (1 frame/s), the tension decrease is slower, limited by how rapidly the cell membrane flows over the cell's surface and into the tether (fig. S2F). Membrane tension recovered more slowly, over a 10- to 20-s time scale (Fig. 4C). The initial rapid decrease in membrane tension was likely due to exocytosis, as no change in cytosolic calcium or membrane tension occurred without nifedipine photolysis or when photolysis was carried out in the absence of extracellular calcium (fig. S12E). No tension changes could be detected in the soma during neuronal activity (Fig. 4D).

The slow recovery of membrane tension was likely due to endocytosis, for two reasons. First, the time scale for membrane tension recovery is within the range expected for slow, clathrin-dependent endocytosis measured using time-resolved capacitance in these cells (*14*, *15*, *31*). Note that the membrane tension recovery here is sufficiently slow that it is unlikely to be distorted by the tether response time (~1 s; fig. S2), unlike the rapid decrease in membrane tension due to exocytosis above. Second, treatment with myristyl trimethyl ammonium bromide (MiTMAB), an agent that blocks endocytosis by inhibiting dynamins I and II (*37*, *38*), inhibited recovery in a dose-dependent manner. The recovered fraction of membrane tension within 50 s of stimulation was ~1.0, 0.49, and 0.29 with 0, 10, or 30 μM MiTMAB, respectively (Fig. 4, C and F, and fig. S12F). We used pro-myristic acid, rapidly broken into myristic acid in the cytosol, as a negative control, which did not affect membrane tension dynamics (fig. S12G). Together, these results demonstrate that SV recycling generates large changes in membrane tension, which are mostly confined to the terminal.

## DISCUSSION

We have shown that cell membranes can flow at vastly different speeds, likely reflecting physiological requirements. In neuronal presynaptic terminals, rapid membrane flow is likely an adaptation required for maintaining SV exo-endocytosis at distinct loci, as recognized nearly 50 years ago (*8*). Because endocytosis is inhibited strongly with increased membrane tension (*4*–*6*), fast terminal-wide equilibration of membrane tension gradients likely contributes to the signaling that couples exo- and endocytosis (*3*, *14*). Conversely, slow membrane flow likely limits the spatial and temporal extent of exo-endocytic coupling in neuroendocrine adrenal cells. Upon fusion, secretory granules rarely collapse into the plasma membrane in such cells (*19*, *20*), and those that do take seconds to do so (*19*, *39*), consistent with the extremely slow membrane flow we observed in chromaffin cells.

Resistance to membrane flow primarily arises from interactions of the cell membrane with the underlying cytoskeleton (*7*, *26*, *40*, *41*). Consistent with hydrodynamic models of 2D flow around fixed obstacles (*7*), we found that tracer mobility is a poor predictor of membrane flow. The fraction of immobile membrane proteins is a better indicator but cannot quantitatively explain the large differences between membrane flows we observed. Tether drag and its sensitivity to agents disrupting the cortical F-actin network correlate much better with the observed membrane flows. Thus, it is likely that membrane tension dynamics are governed not only by the density of the immobile obstacles but also by their spatial arrangements (*42*, *43*), hydrodynamic drag between the membrane and the cytoskeleton, and how easily the immobile obstacles can be detached from the cytoskeleton under flow (*26*, *44*). Like chromaffin cells (*45*), bipolar cell terminals have a dynamic F-actin cortex (*29*, *30*), but the ultrastructural arrangement of the actin cytoskeleton, how it is linked to the PM, and how these interactions affect membrane flows at presynaptic terminals will need to be addressed in the future.

## MATERIALS AND METHODS

### Cell and tissue preparation

All procedures for animal care were carried out according to Yale Animal Care and Use Committee. Goldfish retinal bipolar neuron dissection and dissociation was carried out following (*46*). Goldfish were first dark-adapted for at least 20 min and then decapitated using a scalpel and promptly pithed. Eyes were removed, hemisected, and placed in oxygenated dissociation buffer containing 120 mM NaCl, 2.5 mM KCl, 0.5 mM CaCl_2_, 1 mM MgCl_2_, 10 mM Hepes, 0.75 mM EGTA, 10 mM glucose (pH 7.4), 260 mOsm (osmolarity was checked with the Precision Systems Micro-Osmette Osmometer). Retina was removed from eyecups and placed in hyaluronidase solution (1100 U/ml) (Sigma-Aldrich, H3884), dissolved in dissociation buffer to remove vitreous for a minimum of 12 min. Retinas were removed from hyaluronidase solution and cut into four to six pieces (each about 3 mm by 3 mm). Retina pieces were digested with papain solution (12.5 mg/ml; Sigma-Aldrich, 76220) for 30 min containing l-cysteine (0.5 mg/ml; Sigma-Aldrich, C7352) added to dissociation buffer. Pieces of tissues were removed from papain, rinsed in dissociation buffer, and kept at 12° to 14°C for 4 to 6 hours. To obtain dissociated retinal bipolar neurons, retina pieces were mechanically triturated using a fire-polished Pasteur pipette and plated onto glass-bottom MatTek dishes coated with poly-d-lysine (MatTek, P35GC-1.5-14-C). Retinal bipolar neurons were visually identified by morphology.

Mouse chromaffin cells from 1-month-old mice (C57Bl6) were cultured following (*47*). Briefly, animals were euthanized using isoflurane overdose followed by cervical dislocation, the abdomen was opened, and both adrenal glands were removed. The medullas were dissected using a stereomicroscope and transferred to a sterile petri dish containing 1 ml of sterile ice-cold Locke’s solution containing 154 mM NaCl, 5 mM KCl, 3.6 mM NaHCO_3_, 5 mM Hepes, and 11 mM glucose. Tissues were disaggregated using papain (60 to 90 UI/ml) for 15 to 20 min at 37°C without shaking. Subsequently, tissues were washed with 800 μl of fresh Locke’s solution and passed through 1-ml and 100-μl pipette tips until the suspension became turbid. Cells were plated onto glass-bottom MatTek dishes coated with poly-d-lysine.

### Reagents and materials

The reagents used in this study are listed in Table 1.

**Table 1.** Reagents used in this study

**Reagent or identifier**	**Source**	**Identifier**
Hyaluronidase	Sigma-Aldrich	H3884
l-Cysteine	Sigma-Aldrich	C7352
Papain	Sigma-Aldrich	76220
CellMask Deep Red	Invitrogen	C10046
FM4-64	Invitrogen	T13320
Fluo-4-AM	Invitrogen	F14201
Carboxyl latex bead 1 μm	Invitrogen	C37274
Carboxyl latex bead 2 μm	Invitrogen	C37278
Carboxyl latex bead 3 μm	Invitrogen	C37281
Nifedipine	Abcam	ab120235
MiTMAB	Abcam	ab120466
Pro-myristic acid	Abcam	ab120476
LatA	Cayman Chemical	10010630
SiR-Actin	Spirochrome	SC001

### Optical tweezers

The setup consists of a PerkinElmer UltraVIEW spinning-disc confocal system with a NikonTE2000 inverted microscope, Yokogawa CSU-X1 scanhead, a Hamamatsu C9100-50 electron-multiplying charge-coupled device (EMCCD) camera, and laser lines for 405, 488, 532, and 640 nm, controlled by Volocity or μManager software (*48*). An OT is generated by focusing a 1064-nm laser beam (Coherent Matrix, 10 W) through a 100×/1.45 oil objective lens (Nikon Plan Apo λ) ~10 μm above the coverslip surface. Samples are moved by a piezoelectric stage (300 μm × 300 μm range, Piezoconcept, France), which is controllable with a joystick, LabVIEW Virtual Instruments, or analog signals from a waveform generator (Agilent, 33522A). A micropipette holder is attached to another programmable three-axis piezo unit (100-μm range per axis; P-611.3 NanoCube, with controller E-727 and Mikromove software, Physik Instrumente, Germany) that is used for programmable motion of the pulling tether. The three-axis piezo stage is mounted onto a Newport manual M-462 Series xyz stage for coarse movement.

Trap stiffness was calibrated using a hydrodynamic flow method, following (*49*). In a closed sample cell, the stage is oscillated with a sine wave, with peak-to-peak amplitude *A*_pp_ and drive frequency *f_d_*, while a bead is trapped with OT. The bead positions are recorded using the EMCCD camera, at rates up to 85 Hz from a small region of interest (ROI) around the bead. The stage velocity *v_d_* is assumed to be equal to the liquid velocity, *v_l_*. Inertial forces are negligible because the Reynolds number is typically small: *R_e_* = (*v_l_* × *D*_bead_ × ρ)/η~10^−6^, with *D*_bead_~1 μm, ρ~10^−9^ pN · s^2^/μm^4^ (1 kg/m^3^), η~10^−3^ pN · s/μm^2^, *v_l_*~1 to 10 μm/s. The bead experiences a hydrodynamic force *f*_hydro_ = γ(*v_b_* − *v_l_*), where *v_b_* is the bead velocity and γ = 6πη*R*_bead_. In the regime *w_d_* · τ ≪ 1, where *w_d_* is the angular frequency of stage oscillation (*w_d_* = 2π*f_d_*) and τ = γ/*k*_Trap_, the amplitude of the bead’s motion is *A*_bead_ = τ*w_d_A*_pp_/2. Thus, *f*_bead_ = *k*_Trap_ *A*_bead_ = γ*w_d_ A*_pp_/2 = *f*_hydro_. Trap stiffness *k*_Trap_ is found from the slope of a linear fit to *A*_bead_ versus *f*_hydro_. Trap stiffness was found to be linear with laser power. Trap stiffnesses used were in the range of 73 to 271 pN/μm. We used carboxylated latex beads of 1.9 ± 0.16 or 3.2 ± 0.19 μm (mean ± SD) diameter (Invitrogen; referred to in text as 2- or 3-μm beads).

For tether force measurements, a bead was trapped in the OT and its zero-force position (*x*_o_, *y*_o_) was recorded for at least 10 s and averaged at a rate of ~14 or 30 frames/s. The bead was then brought into contact with the cell surface for ~1 s and then pulled away from the membrane by joystick control of the piezo stage to form a membrane tether. Typically, more than half of attempts were successful with bipolar cells, but with chromaffin cells, the success rate was somewhat lower. Bead positions (*x*, *y*) were recorded using digital image stacks. The presence of a tether was confirmed by visual assessment when possible (e.g., when the cell membrane was labeled with a dye or when the tether was visible in contrast-enhanced bright-field images) or by releasing the bead at the end of the experiment and observing it being pulled back to the cell surface. Bead positions were tracked offline using a custom-made MATLAB (The MathWorks Inc., Natick, MA) program that uses the Image Processing Toolbox function “imfindcircles” to detect the bead using a circular Hough transform algorithm and then calculates the weighted centroid of the bead. The deviation of the bead’s position $\Delta r=\sqrt{{\left(x-x_{o}\right)}^{2}+{\left(y-y_{o}\right)}^{2}}$ from its zero-force position was calculated for each frame and used to calculate the force acting on the bead, *f_b_* = *k*_Trap_Δ*r*, using the trap stiffness calculated as above. Imaging beads immobilized on a coverslip indicated that the centroid of a bead can be detected with a root mean square error of ~17 nm at 33-Hz sampling in bright-field images. With a typical *k*_Trap_ = 75 pN/μm, this translates to an error in force estimation of δ*f_b_* = 1.3 pN. Membrane tension was estimated from the tether force acting on the bead (*26*, *50*), $f_{t}=2\pi \sqrt{2\kappa \;\sigma_{t}}$, where σ is the membrane tension, and κ = 0.27 pN · μm is the membrane bending modulus for neuronal growth cones (*50*), a value within the range reported for other cells (0.18 to 0.32 pN · μm) (*51*–*54*).

### Double-tether pulling

Goldfish retinal bipolar neurons were dissociated and plated into Ringer’s solution without Ca^2+^ [130 mM NaCl, 4 mM KCl, 1 mM MgCl_2_, 10 mM Hepes, 10 mM glucose, 4 mM EGTA (pH 7.3), 260 mOsm]. Chromaffin cells were plated into Locke’s solution lacking Ca^2+^. Calcium was omitted to prevent secretion that could affect membrane tension measurements. A micropipette with a ~1-μm-diameter tip was attached to a pipette holder and lowered into the solution while applying positive pressure. Beads (3 μm diameter) were added to the dish, and a bead was caught at the tip of the pipette by applying negative pressure. Another bead was captured with the OT, and a tether (the probe tether) was pulled to a few micrometers in length and held stationary. The bead held by the micropipette was then lowered further and used to pull a second tether (the pulling tether) from the cell surface to a few micrometers in length using a joystick. After equilibration, the pulling bead was extended by 40 μm at 1 μm/s, held for 30 s, and then returned to the initial position at 1 μm/s using an automated protocol. Images were acquired at a rate of ~13 to 14 or 30 frames/s, except for experiments shown in fig. S5 that used CellMask Deep Red fluorescence to track membrane tension changes, where the frame rate was limited to ~4.2 frame/s due to photobleaching.

### Photostimulation

For photostimulation, goldfish retinal bipolar neurons were dissociated and plated into a glass-bottom MatTek dish using 2 mM Ca^2+^ Ringer’s solution [125 mM NaCl, 4 mM KCl, 1 mM MgCl_2_, 2 mM CaCl_2_, 10 mM Hepes, 10 mM glucose (pH 7.3), 260 mOsm] supplemented with 1 μM Fluo-4-AM, a fluorescent calcium indicator dye, and 20 μM nifedipine, a photolyzable L-type calcium channel blocker. While allowing 30 min for attachment and dye uptake, a bipolar cell was visually identified. Immediately after washing with 2 mM Ca^2+^ Ringer’s solution without nifedipine or Fluo-4-AM, a membrane tether was pulled from the presynaptic terminal using a 3-μm-diameter carboxylated latex bead. Images were recorded alternating between bright field (to record the bead position) and fluorescence (488-nm excitation, 0.5% laser power, Chroma 527/55-nm band-pass emission filter, for calcium imaging using Fluo-4) at a rate of 1 frame/s per channel. For stimulating calcium entry and secretion, nifedipine was photolyzed using the 405-nm laser at full power for 1 s. Bead displacements were analyzed to determine membrane tension as above, and calcium dynamics were analyzed by plotting the fluorescence (background-corrected pixel values in an ROI) relative to its initial value, Δ*F*/*F*_o_.

### Tether sliding

Goldfish retinal bipolar neurons were dissociated and plated into Ringer’s solution without Ca^2+^ [120 mM NaCl, 4 mM KCl, 1 mM MgCl_2_, 10 mM Hepes, 10 mM glucose, 4 mM EGTA (pH 7.3), 260 mOsm] and with 5 μM CellMask Deep Red (Thermo Fisher Scientific) to visualize tethers. A few initial experiments used 14 μM FM4-64 (Invitrogen) instead of CellMask Deep Red, with no obvious difference in results. Tethers were extruded as above using a 2- or 3-μm-diameter bead, either from the terminal or soma of a retinal bipolar neuron or from a chromaffin cell. In some cases, 20 μM LatA (Cayman Chemicals) was added to the bath during cell plating to disrupt the F-actin cytoskeleton.

#### Image analysis

Tether sliding was quantified using a pipeline incorporating semiautomated image processing in ImageJ and analyses in MATLAB. Bead force measurements were performed as described above, using a custom MATLAB script that tracked the bead center through every frame using the Computer Vision toolbox. Videos were registered in the frame of the cell using ImageJ’s Linear Stack Alignment with SIFT plugin (*55*). Significant registration drift was corrected with a manually tracked stationary landmark. The membrane contour was initiated manually for an initial frame, which was then tracked for that and subsequent frames using the JFilament plugin (*56*). The bead center was additionally tracked in the new reference frame. The point at which the bead contacts the membrane (the tether base) was manually tracked through each frame in ImageJ.

#### Data analysis

Points from the tracked cell outline were fit with a spline, and the tangent line was calculated at the nearest point to the tether base. The angle between the line perpendicular to the tangent and a straight line connecting the tether base to the bead defines the tether membrane angle θ. The tangential force on the membrane *f*_∥_ is the sine of the tether membrane angle θ multiplied by the total tether force at the given frame. The cumulative distance traveled by the tether base was extracted from each video. Cumulative distance and force traces were smoothed with a Gaussian with width 1 s. The tether velocity *v_t_* is then calculated per frame as the derivative of the cumulative distance trace.

Forces and velocities were collated across individual experiments within each cell type for further analyses. The maximum supported force (fig. S10E), *f*_∥, max_, is the peak of the force trace when the tether is stalled, i.e., when velocity is below 500 nm/s, estimated to be the level of noise for a stationary tether. Obstacles result in tether base stalling (*v_t_* < 500 nm/s) despite sustaining a substantial tangential force.

### Tracer diffusion and immobile fraction of membrane proteins

Retinal bipolar neurons and chromaffin cells were labeled with Alexa Fluor 488-NHS dye (250 μg/ml; Thermo Fisher Scientific) for 30 min at room temperature (RT) or 37°C. Photobleaching was performed using a Leica SP8 inverted confocal microscope by scanning the 488- and 480-nm beams operating at 100% laser power over a circular ROI (diameter of 2.4 μm) in the imaging plane, set around the middle of the cell’s height. With an optical section thickness of ~0.85 μm, the cell membrane area that is bleached is approximately a 2.4 μm–by–0.85 μm rectangle (in the *xz* plane). Four frames were acquired at low (2%) laser power for normalization of the fluorescence signal before bleaching the ROI for 13 s as explained above. Recovery was monitored for 30 frames every 1290 ms and then 15 to 30 frames every minute at low laser power (2%). Image size was 512 × 512 pixels. The background was determined with the same ROI area outside the cell using ImageJ and subtracted from the signal.

Postbleach fluorescence recovery traces were fit to *F*(*t*) = [*F*_0_ + *F*_∞_(*t*/*t*_1/2_)]/(1 + *t*/*t*_1/2_), where *F*_0_ is the initial postbleach fluorescence, *F*_∞_ is the asymptote, *t*_1/2_ is the half-time of recovery, and *F* is the normalized intensity, *F* = (*f* − *f*_o_)/ *f*_pre_ − *f*_o_), where *f* is the raw intensity, and *f*_pre_ and *f*_o_ are the raw prebleach and postbleach intensities, respectively. Best-fit values for these parameters were estimated using MATLAB’s Curve Fitting Toolbox. The tracer diffusion constant is calculated (*57*) as *D_t_* = *A*_bleach_/(4 *t*_1/2_), where *A*_bleach_ = 2.4 × 0.85 μm^2^ is the photobleached area. The mobile fraction of proteins is estimated as *F*_∞_ − *F*_0_.

### Tether force as a function of tether extension

Goldfish retinal bipolar neurons were dissociated and plated into Ringer’s solution without calcium. A 3-μm-diameter bead was used to pull a short membrane tether as above. After the tether was force-stabilized, it was extended by moving the cell away from the OT center via programmable movement of the piezoelectric stage at 1 μm/s. Images were acquired at a rate of 13 to 15 frames/s, with *k*_Trap_= 73 to 75 pN/μm. Changes in tether force or normalized membrane tension versus extension curves were plotted (fig. S2). For chromaffin cells, a similar protocol was used, except a larger trap stiffness (*k*_Trap_= 271 pN/μm) had to be used to prevent the bead from escaping the trap prematurely.

A sudden short extension of a static tether was used to test the response of the tether force to a step-like tether extension. After a tether was extruded, the tether force was allowed to stabilize for ~10 s. The stage was moved by 1 to 3 μm at 10 to 500 μm/s, while the force was monitored. Force profiles were normalized to $\tilde{f}=(f-f_{0})/(f_{\text{max}}–f_{0})$ and averaged (fig. S2). A double-exponential function was fit to the data using the MATLAB Curve Fitting Toolbox.

### Fluorescence-based tether force estimates

To test the relationship between the tether force *f_t_* and tether diameter *r_t_*, cells were incubated with 5 μM CellMask Deep Red dye for 15 min in calcium-free Ringer’s solution. A short tether was extruded in bright-field mode and held stationary until the force settled. Then, the tether was extended at 1 μm/s, while fluorescence images were acquired at ~4.2 frames/s. Tether force was calculated from the fluorescence channel images as above (CellMask also labeled the beads, allowing their tracking). Tether fluorescence was estimated from an ROI (see fig. S5 for an example). The number of pixels in the tether ROI was doubled using bicubic interpolation and rotated, and the pixels along the direction of the tether were averaged. A Gaussian function was fit to the averaged lateral intensity profile, and the area under the Gaussian was taken as the background-corrected tether fluorescence intensity, *I_t_*. This procedure was repeated for every frame in a movie stack. Typical tether diameters are much smaller than the optical sectioning (~0.85 μm); thus, tether fluorescence is integrated over the thickness of the tether. Because $f_{t}\sim 1/r_{t}\sim \sqrt{\sigma_{t}}$ [see above and (*26*, *50*)] and *I_t_*~2π *r_t_ l*, where *l* is the length of the ROI along the tether, *f_t_*~1/*I_t_*, and $\sigma_{t}\sim 1/I_{t}^{2}$. We plotted the relationship between *f_t_* and 1/*I_t_* in both dynamic experiments (where the tether was being pulled at 1 μm/s; e.g., fig. S5, A and B) and static measurements (where the tether was held at stationary length; fig. S5C). In both cases, a linear relationship was obtained.

To test how membrane tension changes at the pulling tether are communicated to the probe tether, we pulled two tethers simultaneously as described earlier, but we labeled the cells using CellMask Deep Red as above, and imaged cells in fluorescence (~4.2 frame/s). We analyzed the fluorescence of each tether as above and plotted $1/I_{t}^{2}\sim \sigma_{t}$ for the two tethers (fig. S5, D and E).

### Imaging of the F-actin cytoskeleton

For live-cell imaging, cells were incubated with 1 to 2 μM SiR-Actin (Cytoskeleton Inc., Spirochrome) for 15 to 30 min in calcium-free buffer, and then a tether was pulled as described above. SiR-Actin fluorescence was monitored using 640-nm excitation and a Chroma 485/60-nm, 705/90-nm band-pass emission filter using spinning-disc confocal microscopy. We could not detect any SiR-Actin fluorescence in the tethers (five, five, and six tethers extruded from the termini and somas of bipolar cells, and chromaffin cells, respectively). Robust SiR-Actin labeling was evident in every case below the plasma membrane in intact regions of the cells (fig. S4).

We also imaged fixed cells labeled with Alexa Fluor 488–phalloidin and confirmed that treatment with LatA did disrupt the F-actin cytoskeleton (fig. S11). Cells were pipetted onto a poly-d-lysine–treated glass slide and incubated for 30 min. Nonadhered cells were washed away with the standard solutions (Ringer’s and Locke’s for bipolar and chromaffin cells, respectively, both lacking Ca^2+^). Cells were then incubated at RT for 30 min in the absence or presence of 20 μM latrunculin. Subsequently, cells were rinsed three times with the standard solutions and fixed with 4% paraformaldehyde in phosphate-buffered saline (PBS) for 20 min and permeabilized with 0.1% saponin in PBS for 20 min. To reduce nonspecific background staining, cells were incubated with 1% (w/v) bovine serum albumin in PBS for 30 min and were then stained with 1:400 Alexa Fluor 488–phalloidin (Invitrogen) in PBS for 20 min at RT. Fluorescence was imaged using the PerkinElmer UltraVIEW spinning-disc confocal system described above, through a 100×/1.45 oil objective (Nikon Plan Apo λ), with the 488-nm excitation laser set at 2% power and a Chroma 527/55-nm band-pass emission filter. Typical exposure times were 200 ms. For quantification of F-actin fluorescence, first an ImageJ active contour fitting algorithm (*56*) was used to detect cell edges in phase-contrast images. In all cases, the snakes were fit as “curves” using the following parameters: α = 100, β = 100, γ = 800, weight = 0.5, stretch force = 100, deform iterations = 0, point spacing = 0.5, and image smoothing = 1.01. Dendrites were excluded from analysis. Mean fluorescence intensity (mean pixel value) was then calculated using a custom-built MATLAB script using the above-calculated snake coordinates dilated to a three-pixel-wide contour and used as a mask. Background was corrected for every cell by subtracting the mean fluorescence of a three-pixel-wide line outside the cell.

### Statistics

Data were analyzed using MATLAB software. Statistical significance was determined by one-way analysis of variance (ANOVA) (fig. S2C), Kolmogorov-Smirnov test (cumulative distribution function comparisons; fig. S10), the Wilcoxon rank sum test (Fig. 1 and fig. S6), or the Student’s two-sided *t* test (fig. S10).

## Supplementary Materials

### This PDF file includes:

Supplementary Text Figs. S1 to S12 Legends for movies S1 to S6 References

### Other Supplementary Material for this manuscript includes the following:

Movies S1 to S6

Supplementary Data and Code

## Notes

View/request a protocol for this paper from *Bio-protocol*.
