# Supplementary material for: Rapid propagation of membrane tension at retinal bipolar neuron presynaptic terminals
Source: Sci Adv. 2022 Jan 5;8(1):eabl4411. doi: 10.1126/sciadv.abl4411 (PMC11580022; doi:10.1126/sciadv.abl4411)
Supplement: Supplementary file 1 — Supplementary Text Figs. S1 to S12 Legends for movies S1 to S6 References [file sciadv.abl4411_sm.pdf]

Science Advances
AAAS

Supplementary Materials for

Rapid propagation of membrane tension at retinal bipolar neuron presynaptic terminals

Carolina Gomis Perez, Natasha R. Dudzinski, Mason Rouches, Ane Landajuela, Benjamin Machta, David Zenisek, Erdem Karatekin*

*Corresponding author. Email: erdem.karatekin@yale.edu

Published 5 January 2022, *Sci. Adv.* **7**, eabl4411 (2022)
DOI: 10.1126/sciadv.abl4411

The PDF file includes:

Supplementary Text
Figs. S1 to S12
Legends for movies S1 to S6
References

Other Supplementary Material for this manuscript includes the following:

Movies S1 to S6
Supplementary Data and Code

Supplementary Text

DETAILS OF MATHEMATICAL MODELING AND SIMULATIONS

Details of modeling work.

Diffusive model of membrane tension propagation: It has long been known that modest differences in the immobile fraction of membrane proteins may cause dramatic differences in membrane flow *(58, 59)*. This is predicted qualitatively by the hydrodynamics of flow around a bed of fixed obstacles: in 2-dimensions (2D), fixed obstacles produce long-range perturbations to the flow field that substantially slow bulk flow with increasing obstacle density *(7, 59)*, even under conditions when tracer diffusion is relatively unimpeded. A model *(7)* in which transmembrane domain (TMD) proteins interact with the underlying cytoskeleton and act as a fixed array of obstacles predicts membrane tension propagates diffusively, with a tension diffusion coefficient $D_\sigma = Ek/\eta$, where E is the membrane stretch modulus, η is the 2D membrane viscosity, and k is the Darcy permeability of the array of obstacles. Assuming obstacles are randomly distributed, the Darcy permeability is a function of obstacle area fraction ϕ and the radius a of obstacles, $k = a^2 f(\phi)$, where $f(\phi)$ is a rapidly-decaying scaling function *(42)*; for $\phi < 0.37$, $\mathrm{k} \approx -\frac{a^2[1+\ln(\phi)]}{8\phi}$. From FRAP measurements of tracer molecules, Shi et al. *(3)* estimated $\eta \approx 3 \times 10^{-3}$ pN $\cdot$ s/μm and $\phi \approx 0.18$. Combined with published estimates of $E = 40$ pN/μm and $a = 2$ nm, the tension diffusion coefficient was estimated to be $D_\sigma \approx 0.024$ μm^2/s for HeLa cells and similarly small for the four other cell types tested. We interpret this as an upper-bound on the speed of tension-diffusion in these cell-types; Shi and coworkers saw no observable tension propagation in HeLa cells over distances of 5 – 15 μm and timescales $\gtrsim 10$ min (which we confirmed, Fig. S3), which combined with modeling suggest membranes cannot flow faster than what is suggested by this value of D_σ (see *(7)*, Figure 2).

Diffusive model cannot explain measured membrane tension dynamics in bipolar neurons: We used numerical simulations to test if this model could reproduce the membrane tension profiles we measured. We estimated the area fraction of immobile obstacles ϕ from fluorescence recovery after photobleaching (FRAP) measurements (Fig. S8), assuming that the immobile fraction measured in those experiments represents transmembrane proteins that interact with the underlying cytoskeleton. Assuming ~25% of membrane area is occupied by transmembrane proteins *(24)*, we estimate $\phi \approx 0.096 \pm 0.005$ terminal, and $\phi \approx 0.15 \pm 0.005$ in chromaffin cells, yielding $k \approx (6.96 \pm 1.06) \times 10^{-6}$ μm^2 and $k \approx (3.06 \pm 2.3) \times 10^{-6}$ μm^2 respectively. With these constraints, and the values for E, η, and a above, $D_\sigma \approx 0.093$ μm^2/s and 0.048 μm^2/s are predicted for the bipolar and chromaffin cells, respectively. Numerical simulations with these values did not produce satisfactory fits to the data for the terminals or somas. Instead, measured profiles more closely followed the predicted tension at the base of the pulling tether, suggesting rapid propagation of membrane tension with a weak distance dependence (Figure 2A, Fig. S9).

Tension propagation in bipolar neurons shows no clear distance dependence. Diffusive mechanisms predict a strong distance dependence in the amplitude and kinetics of a propagating signal. We explored the distance dependence of the kinetics and amplitude of membrane tension perturbations further to determine if the diffusive propagation model proposed by Shi et al. *(7)* is applicable for bipolar neurons. We plotted the time lag, t_{sense}, between the onset of the tension increase at the pulling tether (i.e., when tether extension was initiated) and the onset at the tension increase at the probe tether in Figure 2B. We similarly plotted the maximum change in

membrane tension at the probe tether, $\Delta\sigma_{max}$, for a given perturbation created at the pulling tether (Figure 2C). To estimate t_{sense} and $\Delta\sigma_{max}$ accurately and in an unbiased manner, we matched a template to the probe tether profile, by shifting it in time and varying its amplitude until a good match was produced. The template was the model predicted tension profile at the pulling tether shifted in time and rescaled in amplitude (see below for details), which, perhaps surprisingly, yielded curves which provided a good description of the experimental tension profiles at the probe tether. Note, however, that any empirical template that matches the experimental profile well could be used to estimate t_{sense} and $\Delta\sigma_{max}$. We found that t_{sense} and $\Delta\sigma_{max}$ obtained in this manner have no clear distance dependence (Figure 2B,C). The time lag t_{sense} as a function of inter-tether distance could only be well-fit with a diffusion constant of ~24 μm^2/s (1000-fold larger than the value estimated *(7)* for Hela cells) or larger, while $\Delta\sigma_{max}$ could not be fit by simply changing the Darcy permeability k to change D_σ (Figure 2B,C). Other membrane parameters may govern tension dynamics. Area expansion modulus measurements are sparse and we take $E = 40\,\text{pN}/\mu\text{m}$, however extreme values up to $E = 1750\,\text{pN}/\mu\text{m}$ have been recorded and suggest substantial cell-to-cell variability *(60)*. Viscosity measurements are also sparse, but have not been found to vary over orders of magnitude. Overall, these results suggest that in bipolar cells membrane tension propagates rapidly and in a qualitatively different manner from its very slow propagation in chromaffin or HeLa cells tested here, or other cell types tested by Shi et al. *(7)*. However, our bipolar cell measurements are consistent with rapid membrane flows reported by Dai and Sheetz *(61)* during axonal growth.

Although it is difficult to rule out that membrane tension may propagate diffusively in bipolar neurons, a diffusive mechanism would require some combination of the physical parameters (viscosity, stretch modulus, Darcy permeability) to be several orders of magnitude different than those measured in other cell-types. Alternative mechanisms could explain rapid membrane flows in bipolar neurons. For example, the model in ref. *(7)* assumes that obstacles are distributed without particular structure and are immobile during the measurements (that span minutes). If the obstacles are arranged differently, rapid membrane flow would be possible at similar obstacle densities, as proposed by Cohen and Shi *(42)*. Other possible mechanisms that could contribute to rapid flows are more dynamic connections between the transmembrane domain proteins (the obstacles) and the underlying cytoskeleton *(26, 62)*, rapid cytoskeleton turnover, three-dimensional propagation of pressure perturbations *(63,64)* or other signaling mechanisms. We hope our results will motivate future modeling and experimental work to better understand cell membrane tension propagation mechanisms.

Numerical methods

Tension Diffusion Simulations. Simulations of tension propagation closely followed those detailed in Shi et al. *(7)*. We simulate the plasma membrane as a disk with a radius $R_{cell} = 100\,\mu m$, divided radially into evenly spaced 0.1 μm concentric circles. We 'pull' an initial tether of length $L_0 = 10$ μm and calculate the tether area A_t, tension σ_t, and radius r_t:

$$r_t = \frac{2\pi\kappa}{f_{t,0}} \quad (1)$$

$$\sigma_{t,0} = \frac{\kappa}{2r_t^2} \quad (2)$$

$$A_t = 2\pi r_t L_0 \quad (3)$$

Where κ is the bending modulus of the membrane and $f_{t,0}$ is the initial tether force. We pull or retract the tether at $\dot{L} = 1$ µm/s or hold it at constant length according to the protocol. The tether radius and tension are updated at constant tether area:

$$L \rightarrow L + \dot{L}dt \quad (4)$$

$$r_t \rightarrow \frac{A_t}{2\pi L} \quad (5)$$

$$\sigma_t \rightarrow \sigma_t + \frac{4\pi\kappa L}{A_t^2} \quad (6)$$

Membrane flows on the cell surface with diffusive dynamics, with diffusion coefficient $D_\sigma = Ek/\eta$. The tension across the membrane is determined as solutions as a boundary-value problem PDE for $\sigma(x,t)$, $\frac{\partial\sigma(x,t)}{\partial t} = D_\sigma \frac{\partial^2\sigma(x,t)}{\partial x^2}$, with tension at $\sigma(0,t) = \sigma_t$ and $\sigma(x = R_{cell}, t) = \sigma_0$ between times t and $t + dt$ using MatLab's partial differential equation solver *PDEPE*. Equations were parameterized in axisymmetric coordinates, where x is the radial distance from the tether base. Solutions to these equations at time t, $\sigma(x,t)$, were used as initial conditions for the subsequent step's calculation. After updating tension across the cell, lipids flow into or out of the tether. The tether's area change is set by the membrane flux at the cell-tether boundary and the tether tension updated accordingly:

$$\delta A = \frac{2\pi r_t k}{\eta} \nabla_{r=0} dt \quad (7)$$

$$A_t \rightarrow A_t - \delta A \quad (8)$$

$$\sigma_t \rightarrow \sigma_t - \frac{2\pi^2\kappa L^2}{A_t^3}\delta A \quad (9)$$

Solutions were calculated iteratively until the completion of the tether pulling protocol: 10 s at constant tether length, 40 s extension at 1 µm/s, 30 s relaxation at constant tether length, 40 s retraction at 1 µm/s, 30 s relaxation. Solutions can be examined at a given inter-tether distance.

Extracting t_{sense} and $\Delta\sigma_{max}$ from experiments with the aid of simulations. Tension measurements from double-tether experiments were smoothed with a Gaussian filter with width set to 1s prior to fitting. We estimated t_{sense}, the time to sense at the probe tether a tension perturbation initiated at the pulling tether at $t = 0$, and $\Delta\sigma_{max}$, the maximum change in tension at the probe tether. Individual tension traces were fit to the equation $g(t) = f(t - t_{sense}) \times \Delta\sigma$ where $f(t)$ is the simulated tension at the pulling tether. We fit two parameters, t_{sense} and $\Delta\sigma$, to each experimental curve by minimizing the mean-squared error between the simulation and experimental curve: $mi = \int dt \left(f_{exp}(t) - g(t)\right)^2$. We extracted $\Delta\sigma_{max}$ from experiments averaging the tension in a half-second window surrounding the maximum recorded

tension in the unfiltered trace. t_{sense} and $\Delta\sigma_{max}$ were calculated similarly from simulated traces to give the curves displayed in Figure 2B,C. Tension traces retrieved from simulation at inter-tether distances of 0.1 – 20 μm are fit to the simulated tension at the tether base to give t_{sense}. $\Delta\sigma_{max}$ was extracted at these inter-tether distances by averaging tension the half-second window of the maximum.

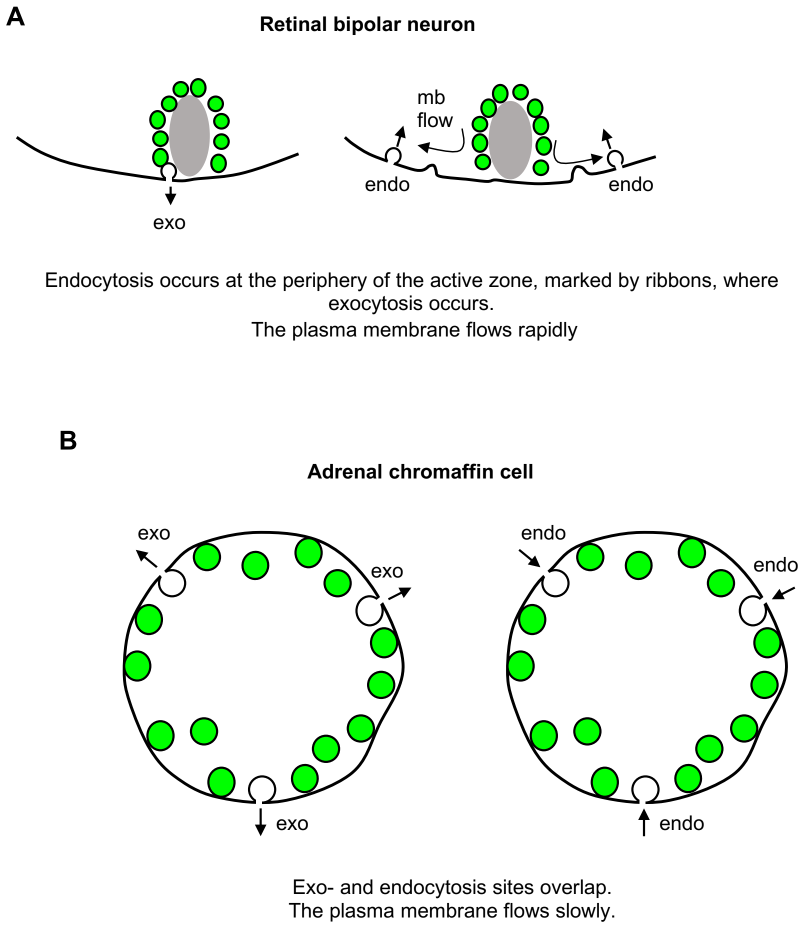

Figure S1. Summary of major findings. **A**. Membrane tension propagates rapidly in the retinal bipolar cell terminal, allowing rapid synaptic vesicle turnover with exo- and endocytosis occurring at distinct loci. **B**. In adrenal chromaffin cells, membrane tension propagates slowly, restricting exo- and endocytosis to occur at overlapping sites.

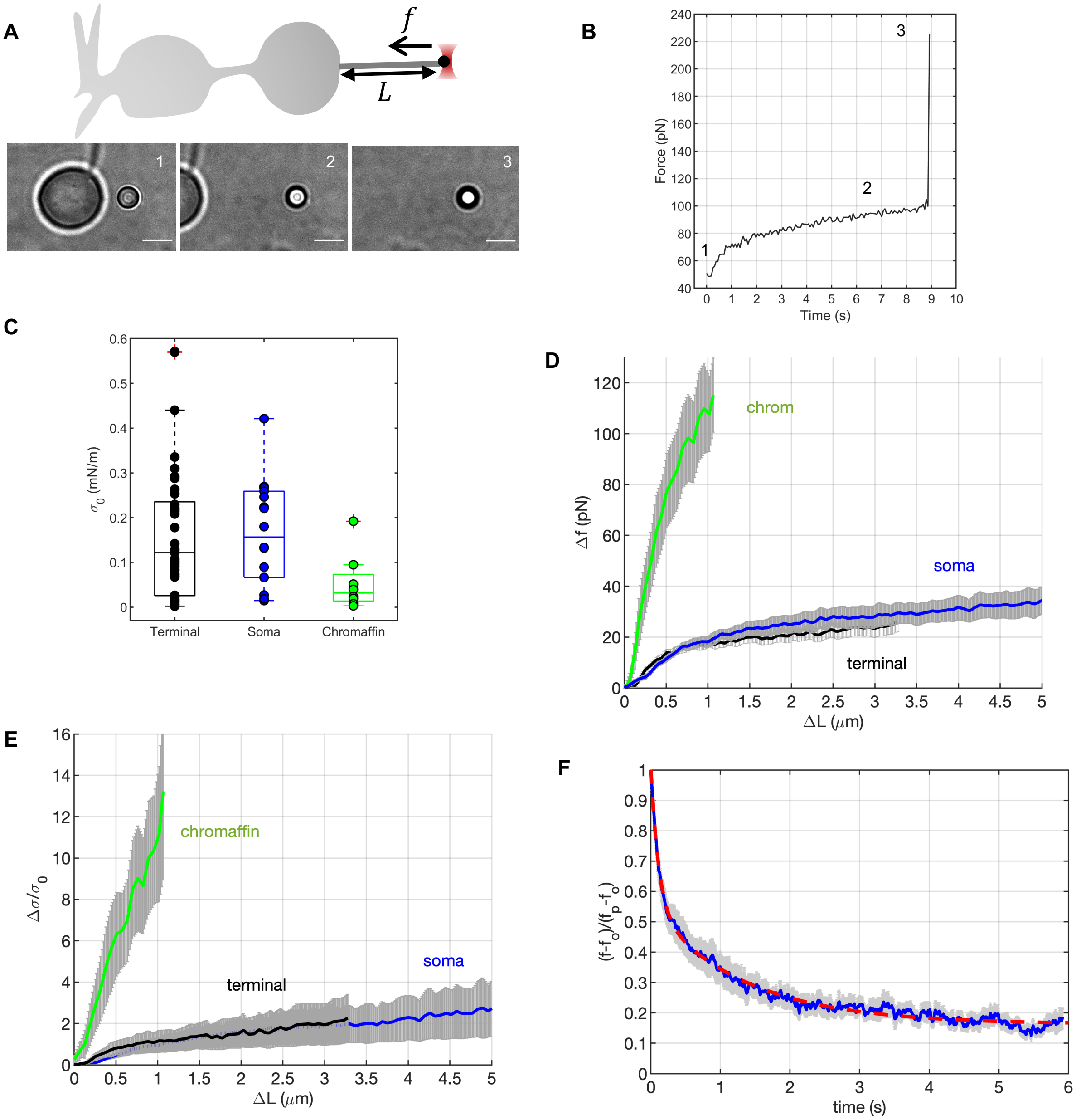

Figure S2. Resting membrane tension and how it changes upon tether extension. A. Top: schematic of the experiment. A 3 μm bead held in the optical trap (OT) is used to pull a short, $L_0 = 1.5 - 3$ μm membrane tether from the cell surface. After the force is stabilized to its static value f_0, the tether is extended at constant speed ($\dot{L} = 1$ μm/s) by moving the cell away while the force acting on the bead $f(t)$ is monitored. Bottom: snapshots of a tether pulled from a bipolar neuronal terminal. Scale bar = 5 μm. **B**. Example of a tether force measurement, for a tether extruded from the terminal for the example shown in **A**. The tether was extended starting $t = 0$ s. The numbers in the snapshots in **A** correspond to the numbers indicated on the force profile. The bead escaped the trap at 3. **C**. Static membrane tension values for bipolar cell terminals (n= 37), somas (n=18), and for chromaffin cells (n=8). There was not a significant difference among the means (p=0.098, 1-way ANOVA). **D**. Change in tether force as a function of tether extension at constant extrusion velocity ($\dot{L} = 1$ μm/s) for chromaffin cells (n=6), or

somas (n= 8) or terminals (n=9) from bipolar neurons. The gray errorbars represent S.E.M. **E**. The force profiles in **D** replotted as the fractional change in membrane tension (using $\sigma_t = f_t^2/(8\pi^2\kappa)$, see Methods) as a function of extension $\Delta L = L - L_0$. **F**. Response of the tether force to a sudden extension. A resting tether, drawn from the terminal, was rapidly extended by 1-3 µm. Shown is the average of the resulting changes in the tether forces, rescaled as $\tilde{f} = (f - f_0)/(f_{max} - f_0)$. The red dashed line is a fit to $\tilde{f}(\mathrm{t}) = \mathrm{a}\ \exp(-\mathrm{t}/\tau_1) + \mathrm{b}\ \exp(-\mathrm{t}/\tau_2) + \mathrm{c}$, with best fit parameters (with 95% confidence bounds): $a = 0.47\ (0.46, 0.48)$, $b = 0.38\ (0.38, 0.39)$, $c = 0.163\ (0.161, 0.164)$, $\tau_1 = 0.112\ (0.107, 0.116)$, $\tau_2 = 1.35\ (1.32, 1.39)$, $R^2 = 0.987$ (n= 9 tethers).

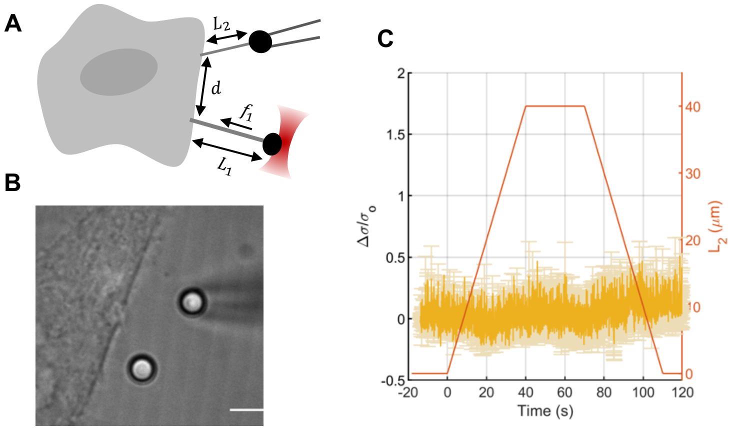

Figure S3. Membrane tension does not propagate in HeLa cells. A. Schematic of the experiment. A "probe" tether is pulled from a HeLa cell using a 3 μm diameter latex bead held in an optical trap (OT). A second, "pulling" tether is extended from the cell using another bead held by a micropipette mounted on a 3-axis programmable piezoelectric stage. While the length L_2 of the pulling tether is extended at 1 μm/s by 40 μm, held for 30 s, then returned to its original position, the probe tether is held stationary, and its tension is measured through the force acting on the bead held in the OT. **B**. A snapshot from an experiment. **C**. Change in membrane tension σ of the probe tether relative to its resting value, σ_0, as a function of time. Average from 6 cells is shown. Error bars indicate standard error of the mean. The length L_2 of the pulling tether is shown on the right-axis (red) . Inter-tether distance d was 4-12 μm. Scale bars: 5 μm.

A B C

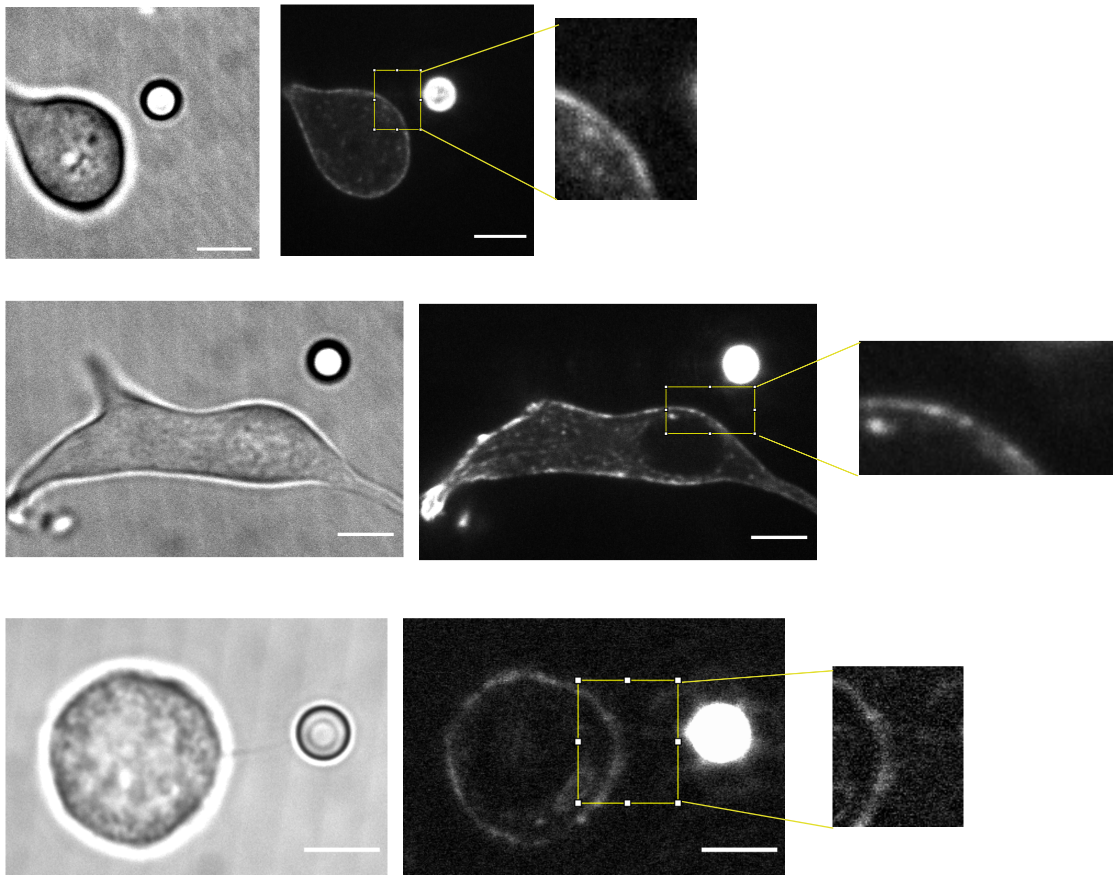

Figure S4. Actin filaments cannot be detected in membrane tethers. A. A tether was drawn from the terminal of a bipolar neuron after SiR-Actin labeling of the F-actin cytoskeleton. Despite good cortical labeling as reported *(29)*, no F-actin is detectable in the tether. The experiment was repeated with 4 other cells, with similar results. **B**. A tether drawn from the soma, with no labeling evident in the tether. Two additional cells were tested, with similar results. **C**. A tether drawn from a chromaffin cell, after Sir-Actin labeling. No F-actin can be detected in the tether. Five other cells were tested, with similar results. Scale bars represent 5 µm.

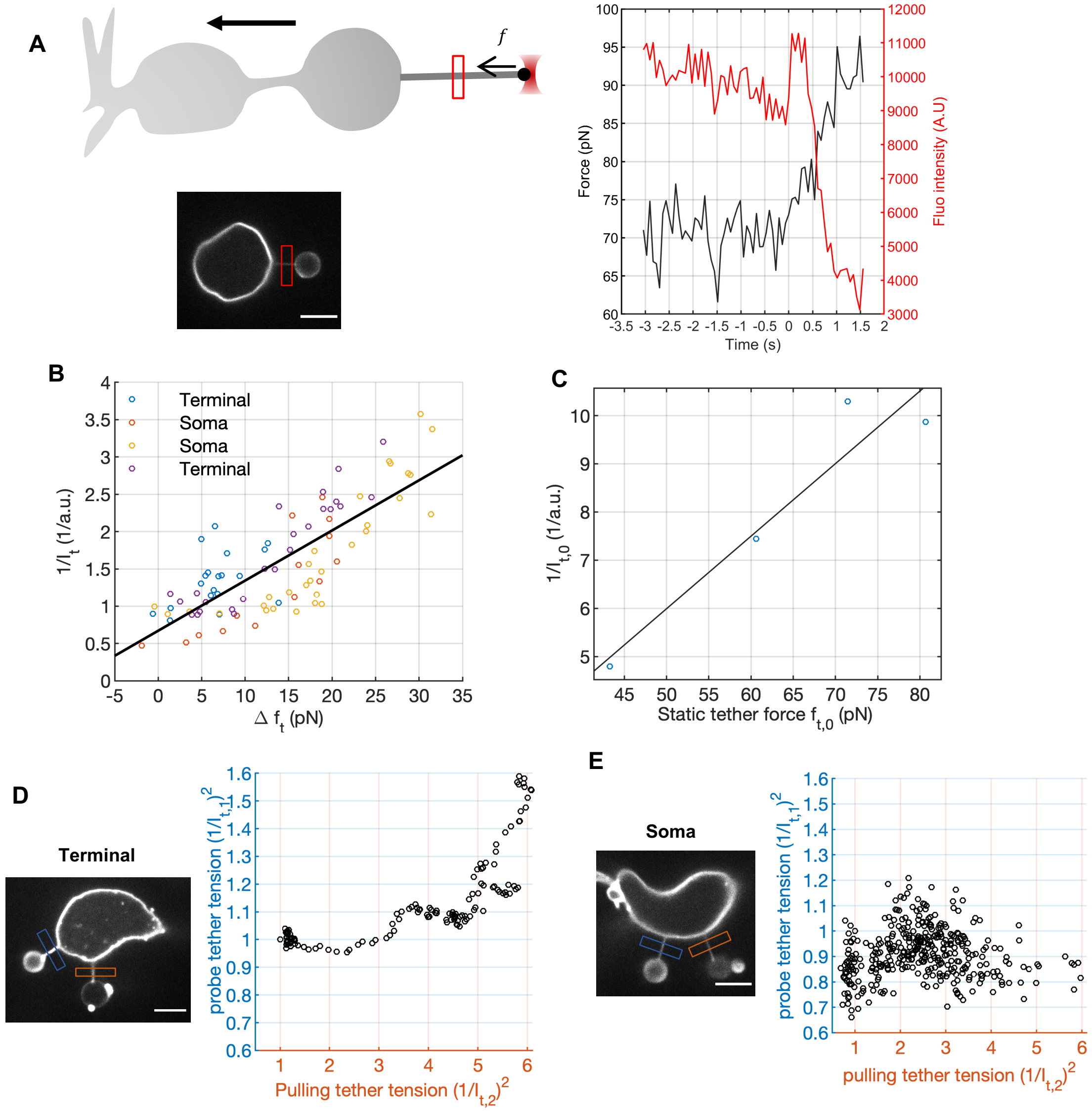

Figure S5. Membrane tension changes tracked using tether fluorescence. **A-C**. Calibration of tether fluorescence as a function of tether force. Cell membranes were labeled with CellMask Deep Red and fluorescence was measured from a fixed area using spinning disc confocal (SDC) microscopy. **A**. Example of a simultaneous force and fluorescence measurement for a tether pulled from the terminal at constant speed (1 μm/s). Left: schematic of the experiment and a snapshot from an image stack recorded while the tether was extended. Right: tether force (left axis, measured using the optical trap) and fluorescence (right axis, from the area marked on the left snapshot) as a function of time. Tether extension started at $t = 0$ s. **B**. Inverse tether fluorescence intensity as a function of the increase in tether force from its initial value. Data from 4 experiments, grouped by color. A linear fit to all data is shown (slope = 6.72×10^{-6} AU/pN, $R^2 = 0.861$). **C**. Relationship between inverse tether fluorescence and tether force for static measurements (e.g., the values for $t < 0$ in **A**). A linear fit had best slope 1.51×10^{-6} AU/pN ($R^2 = 0.910$). **D,E**. Measurement of tether fluorescence simultaneously from two tethers drawn from the same cell. The probe tether (blue box) is held stationary while the pulling tether (red box) is extended at $\dot{L} = 1$ μm/s. The square of the inverse tether fluorescence

intensity ($(1/I_t)^2$) is proportional to membrane tension, since the tether force $f_t \sim 1/r_t \sim \sqrt{\sigma}$ and $f_t \sim 1/I_t$ (see B, C). The regions of interest marked with blue (probe tether) and red (pulling tether) boxes were used to calculate the fluorescence intensities (see Methods), which were normalized to their initial values. Scale bars are 5 μm.

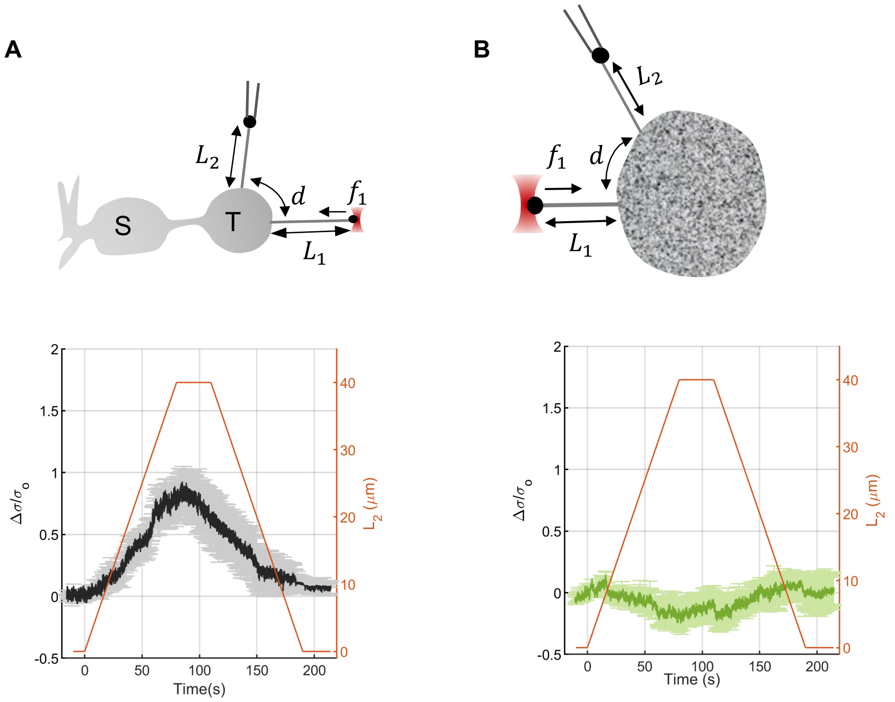

Figure S6. Membrane tension propagation tested while moving the pulling tether at slower speed. A. Top. Schematic of the experiment. These experiments were similar to those shown in Figure 1 except the pulling tether was moved 2-fold slower at $\dot{L} = 0.5\ \mu m/s$. Bottom: Change in probe tether membrane tension relative to its resting value. The average of 5 measurements is shown, with inter-tether distances $6 - 12\ \mu m$. Error bars indicate standard error. **B**. Same as in A, but for neuroendocrine chromaffin cells. Average of 5 measurements, with inter-tether distances $7 - 10\ \mu m$. The medians of the maximal values of $\Delta\sigma/\sigma_0$ are different for bipolar cell terminals and chromaffin cells ($p<0.05$, Wilcoxon rank sum test). Scale bars represent 5 μm.

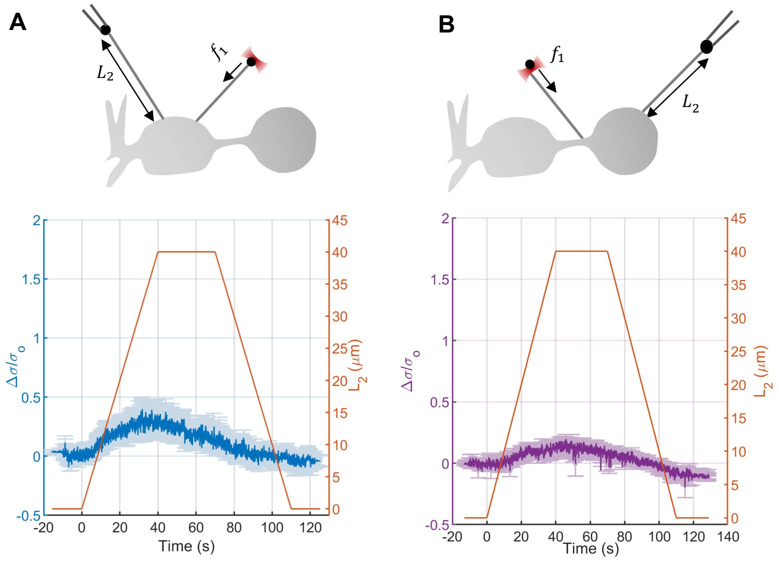

Figure S7. Propagation of membrane tension within the soma and between the terminal and the axon. **A**. Membrane tension change in the probe tether relative to its initial value (left axis) as a function of time as the pulling tether is extended, held stationary, then relaxed (right axis), for tethers pulled from the soma of bipolar neurons (n = 8). Inter-tether distance d was 4−12 μm. **B**. Similar measurements, but the pulling tether was extruded from the terminal, while the probe tether was placed in the axon, with inter-tether distances 14-17 μm (n = 4).

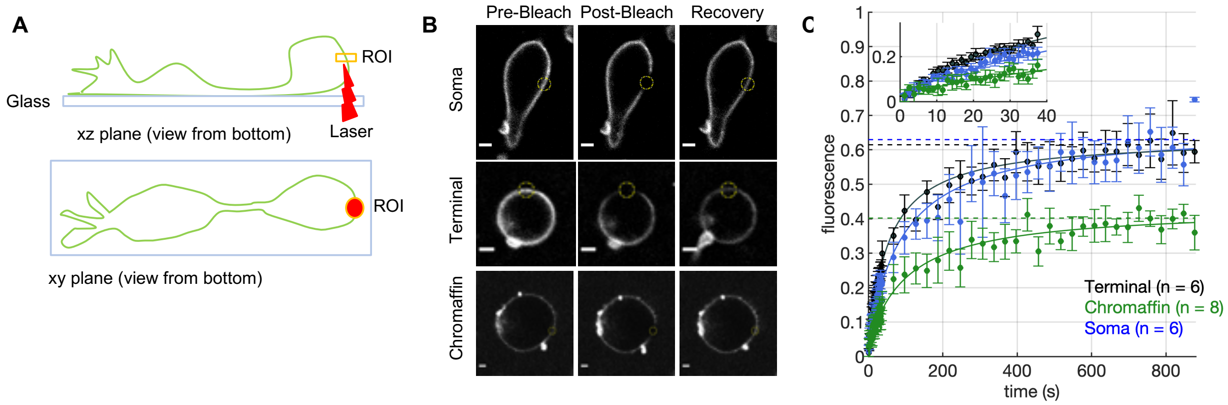

Figure S8. Diffusion and immobile fraction of membrane proteins. A. Schematic of the fluorescence recovery after photobleaching (FRAP) measurements for a bipolar cell terminal. Membrane proteins on the cell surface were labeled with Alexa-488 (see Methods). After washing unreacted dye, a 2.4 µm diameter circular region-of-interest (ROI) was bleached in a single optical slice (~0.84 µm thick) in the middle of the terminal. The resulting bleached membrane area is a ~0.84 µm × 2.4 µm rectangle in the xz plane. **B**. Snapshots from actual measurements for a chromaffin cell, a bipolar neuron soma and a terminal (cells are imaged from the bottom). Scale bars are 5 µm. **C.** Fluorescence recovery in the bleached regions was followed as a function of time. The intensity of the ROI for 4 frames prior to bleaching was averaged and used for normalization of the pre-bleach intensity to 1. Recovery was followed initially at 0.773 frames/s for 30 frames, then at a lower rate (0.25-0.50 frames/s) for the remainder of the measurements to minimize photobleaching while capturing the initial rapid phase. Recovery traces for every group were fit to an equation, with the mobile fraction of protein ϕ_m and the tracer diffusion coefficient D_t estimated from the best-fit parameters (see Methods). Best fit values were (with 95% confidence intervals): $D_t = (10 \pm 7) \times 10^{-3}$, $(19 \pm 6) \times 10^{-3}$, and $(11 \pm 5) \times 10^{-3}$ µm^2/s, and $\phi_m = 0.60 \pm 0.03$, 0.39 ± 0.02, and 0.37 ± 0.04 for chromaffin cells, termini, and somas, respectively. The dots represent experimental measurements. Inset shows the initial recovery on an expanded scale.

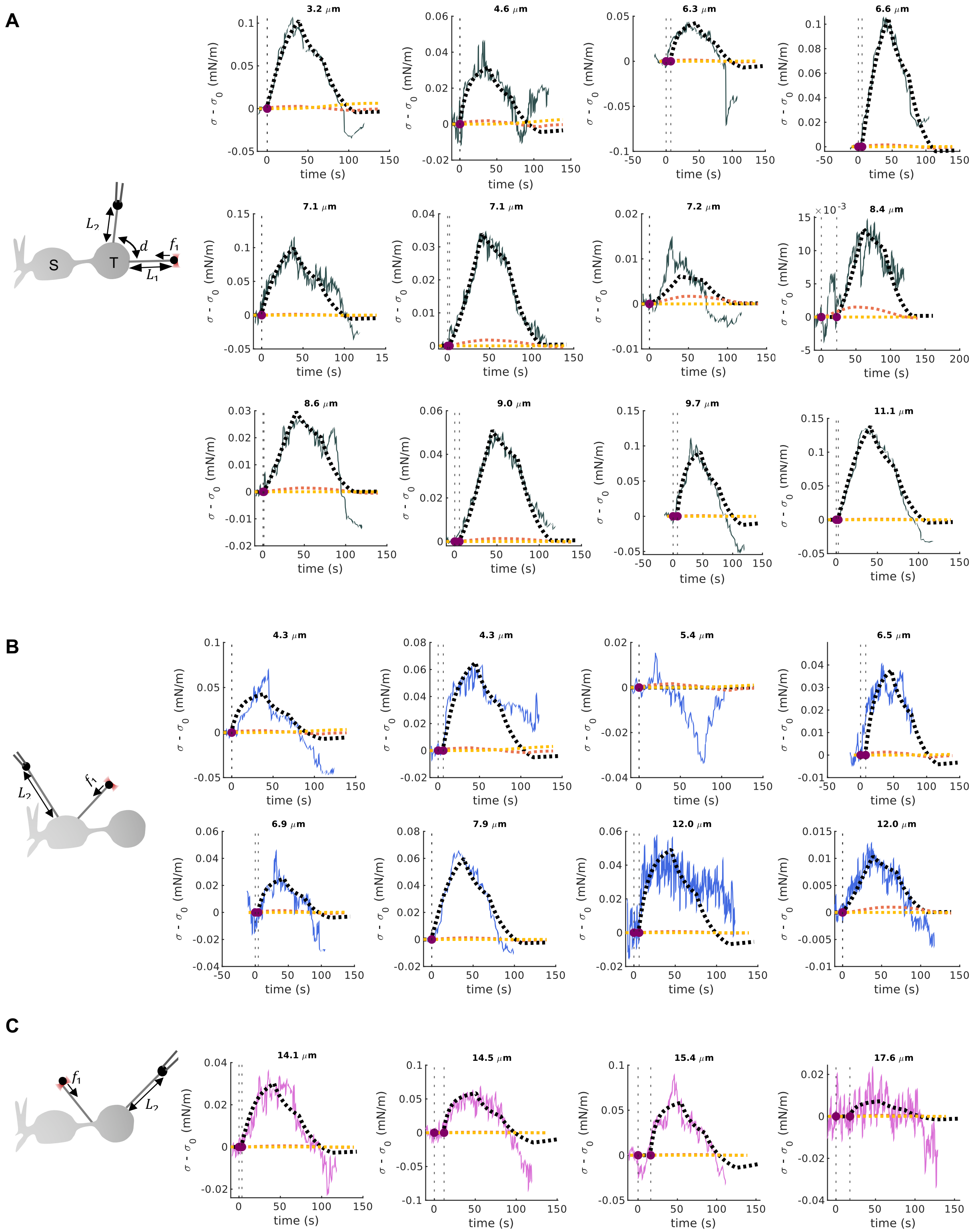

Figure S9. Comparison of experimental measurements of membrane tension propagation with numerical simulations. A. A pulling tether was extended from a bipolar cell terminal by 40 μm at 1 μm/s, held for 30 s, then relaxed to its initial extension at 1 μm/s while a probe tether measured the resulting membrane tension changes a distance *d* away. Left, schematic of the experiment. Right: individual measurements of membrane tension changes (black, solid), shown

with the predicted tension changes at the probe tether assuming a membrane tension diffusivity $D_\sigma = 0.024$ μm²/s (yellow, ref. *(7)*) or a 100-fold larger value (orange). The model *(7)* considers membrane flow through a random array of immobile obstacles (transmembrane domain proteins attached to the underlying cytoskeleton) and predicts diffusive propagation of membrane tension perturbations. The intermembrane distance d for every experiment is indicated. Agreement between simulations and measurements is poor in every case. A good match is obtained only assuming $d = 0.1$ μm (black, dashed), suggesting the tension perturbation created by the pulling tether is transmitted with little distance dependence to the probe tether (see Fig. 2). **B.** As in **A**, but both tethers were extruded from the soma (blue traces) of bipolar neurons. **C**. As in **A**, but the pulling and probe tethers were extruded from the terminal and the axon, respectively (purple traces).

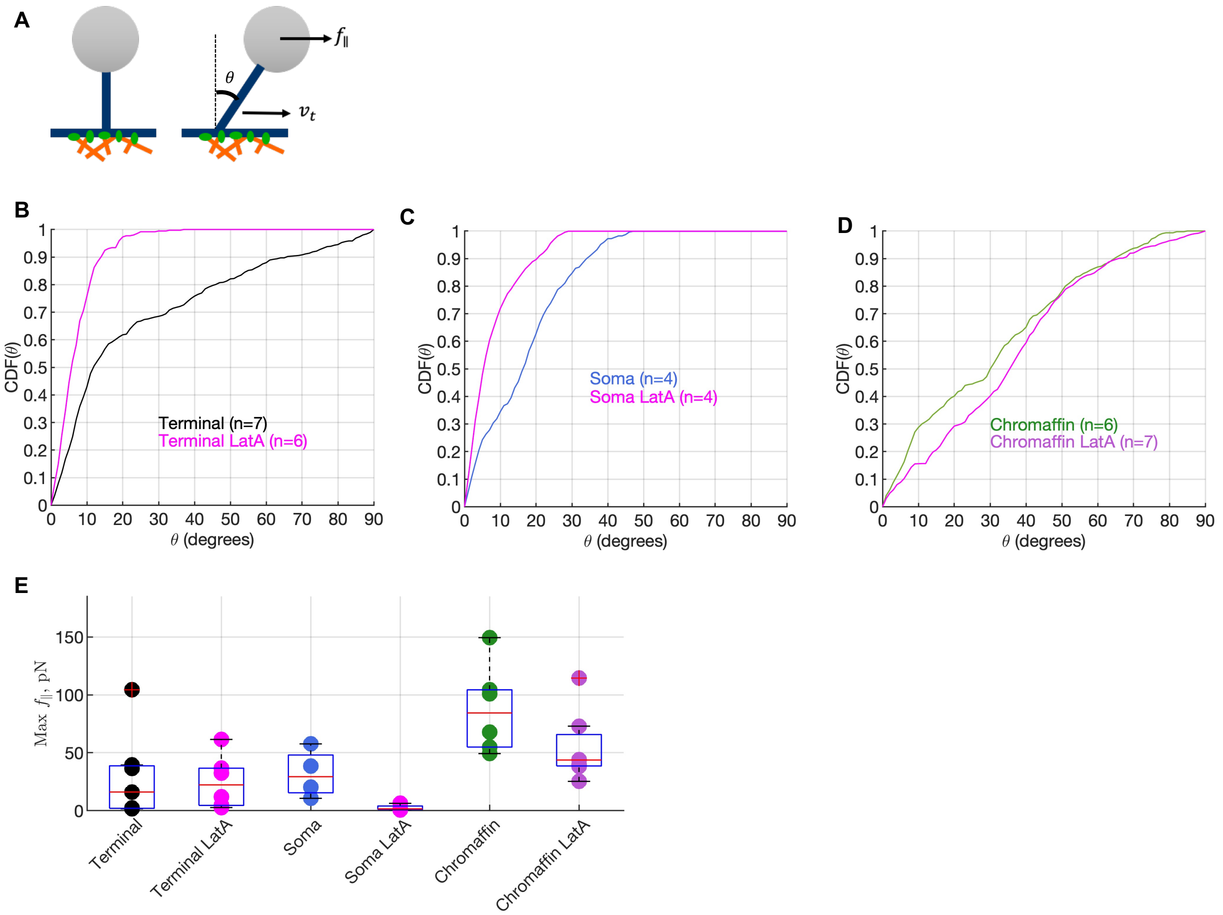

Figure S10. Analysis of membrane tether sliding experiments probing cytoskeleton-membrane friction, related to Figure 3. A. Schematic of the experiment. A membrane tether was pulled from the cell surface, then the cell was moved (by moving the xy stage) to create a tangential force $f_\parallel$ to drive sliding of the tether's base with velocity v_t (see Figure 3 and Methods for details). **B**. Cumulative distribution function (CDF) of tether-membrane angles for bipolar cell terminals. Angles were smaller for LatA-treated neurons, indicating easier sliding and lower forces. **C**. Similar measurements for tethers drawn from bipolar cell somas. **D**. Same as in C, for chromaffin cells. Angles were smaller for terminals and somas compared to chromaffin cells, indicating easier sliding. LatA treatment shifted θ values to lower values in terminals and somas, but not in chromaffin cells (Kolmogorov-Smirnov test, $p < 0.01$, for treated vs. untreated terminals, $p < 0.05$ for somas, and $p = 0.38$ for chromaffin cells, respectively). **E**. Maxiumum supported tangential tether force for bipolar cell terminals and somas, and chromaffin cells. Obstacles result in tether base stalling (during which $v_t < 0.5$ μm/s) despite being subject to a substantial tangential force ($f_\parallel > 5$ pN). Maximum force sustained during such stall periods are plotted (p < 0.05 for Terminal - Chromaffin, Soma - Chromaffin, and treated vs untreated Soma comparisons (Student's 2 sided t-test).

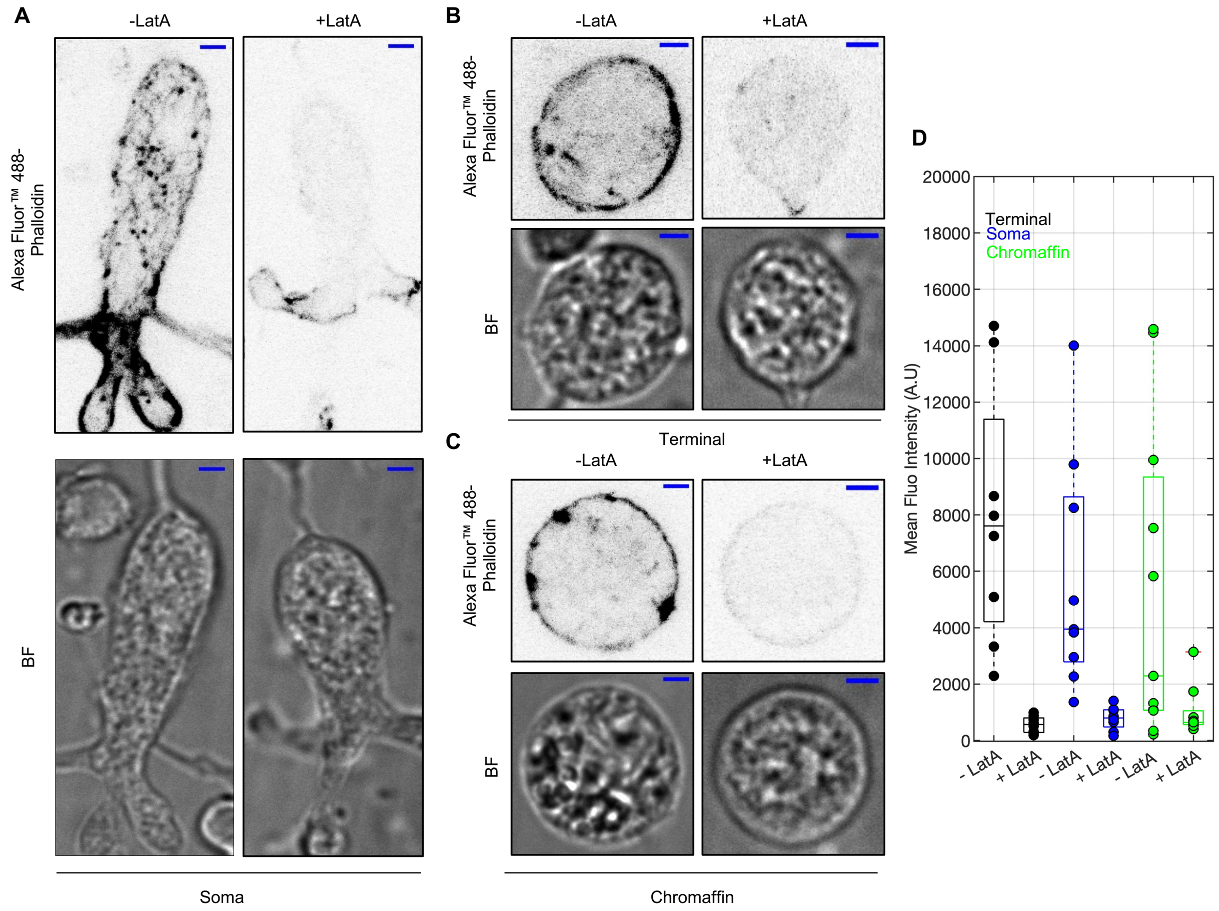

Figure S11. Latrunculin A disrupts the F-actin cortex in bipolar neurons and chromaffin cells. **A-C.** Representative images of F-actin staining with Alexa Fluor 488 Phalloidin (upper panels) and brightfield (BF, lower panels) from a bipolar cell soma (A), a terminal (B) and a chromaffin cell (C) incubated in the absence (-LatA) or the presence (+LatA) of 20 µM Latrunculin A for 20 min at room temperature. Fluorescence images are displayed with an inverted look-up table (stronger staining appears darker). Scale bars are 2 μm. **D**. Background-corrected cell contour mean fluorescence intensities of Alexa Fluor 488 Phalloidin signals for the indicated cell types. Each point represents one cell.

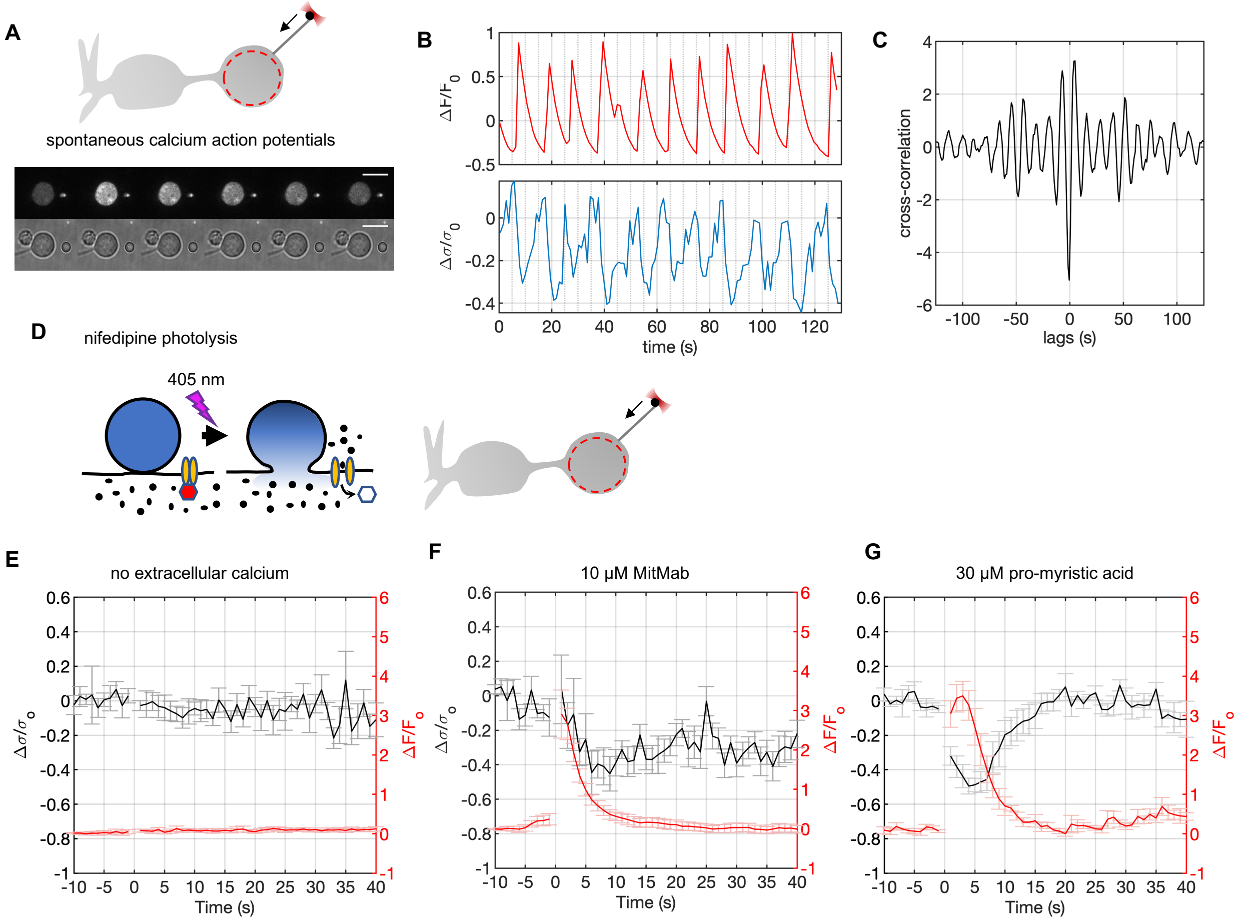

Figure S12. Additional experiments probing the relationship between synaptic vesicle recycling and membrane tension changes. A. Top. Schematic of experiments relating spontaneous exo-endocytic activity of bipolar neurons to membrane tension changes in the terminal. A tether was extruded from the terminal using a bead held in an optical trap to monitor membrane tension. The cell was pre-loaded with a fluorescent calcium indicator (Fluo-4) whose fluorescence intensity was monitored in the region of interest (ROI) indicated by the dashed circle in the terminal. Two mM calcium was present in the extracellular medium. Some cells develop spontaneous calcium action potentials (monitored via Fluo-4 fluorescence) that robustly drive exo-endocytosis *(22)* Bottom: Snapshots from an image stack of a cell that developed spontaneous calcium activity. Brightfield and fluorescence channels were alternated (1.08 s between pairs of images). Scale bar=5 μm. **B**. Quantification of the Fluo-4 calcium signals and membrane tension measurements for the example shown in **A**. Both the fluorescence (top, red) and membrane tension (bottom, blue) are reported as changes relative to the initial value ($\Delta F/F_0$ or $\Delta\sigma/\sigma_0$). Exocytosis occurs with the upstoke of the calcium wave, followed by endocytosis restoring membrane area *(22)*. Note that calcium variations anti-correlate with membrane tension changes, consistent with exocytosis lowering and endocytosis restoring membrane tension, respectively (see Movie S6). Five other cells developed spontaneous calcium activity during membrane tension measurements; all such cells also displayed anti-correlated membrane tension variations. **C**. Cross-correlation between calcium signals and membrane tension for data shown in **B**. Notice the strong negative correlation between the two signals at lag ≈ -0.5 s, the time lag between successive fluorescent (calcium) and bright field (membrane tension)

measurements, indicating membrane tension decreases immediately after a calcium increase with our time resolution. **D**. Schematic of the photostimulation protocol (see Figure 4). Calcium signals were integrated from the ROI shown as a dashed red line in the terminal. **E**. When extracellular calcium was omitted, nifedipine photolysis caused changes neither in calcium signals nor in membrane tension. **F.** When endocytosis was inhibited with 10 μM dynamin inhibitor MiTMAB, calcium signals and the initial decrease in membrane tension were not affected, but the recovery of membrane tension was compromised (cf. Figure 4C). **G**. We used pro-myristic acid as a negative control for the MiTMAB experiments. In the cell, pro-myristic acid is rapidly converted to myristic acid which is not a dynamin inhibitor *(37)*. Membrane tension and calcium dynamics were not appreciably altered compared to controls (Figure 4C). In F and G, 2 mM extracellular calcium was present.

Movie S1. Related to Figure 1C. Testing propagation of membrane tension in a bipolar neuronal terminal using a double-tether protocol. Frames were acquired at ~33 Hz. Scale bar is 5 µm.

Movie S2. Related to Figure 1E. Testing propagation of membrane tension in a chromaffin cell using a double-tether protocol. Frames were acquired at ~17 Hz. Scale bar is 5 µm.

Movie S3. Image stack showing sliding of a membrane tether drawn from the terminal of a bipolar neuronal terminal. The cell membrane was labeled with the lipophilic dye FM4-64. Elements of the tracking, the bead position, position of the tether- base, membrane contour, and tether-membrane tangent line, are overlayed in magenta. Images collected at 8.77 frames/s. The tether slides freely along the membrane. Related to Figure 3.

Movie S4. Image stack showing attempt to drag a membrane tether drawn from a chromaffin cell whose membrane was labeled with CellMask Deep Red. Elements of the tracking, the bead position, position of the tether base, membrane contour, and tether-membrane tangent line, are overlayed in magenta. Images collected at 4.34 frames/s. The tether did no slide along the membrane despite application of tangential forces. Related to Figure 3.

Movie S5. Image stack showing tether sliding in a retinal bipolar neuron soma. The membrane was labeled with CellMask Deep Red. Elements of the tracking, the bead position, position of the tether base, membrane contour, and tether-membrane tangent line, are overlayed in magenta. Images collected at 5.55 frames/s. The tether slides briefly after a tangential force is applied, but becomes stuck again. Related to Figure 3.

Movie S6. Related to Fig. S12A-C. Image stack showing spontaneous intracellular calcium variations anti-correlate with membrane tension changes in a bipolar neuronal terminal. Two channels were alternated during acquisition: brightfield (to detect bead position for membrane tension estimation, 30 ms exposure) and fluorescence (to detect calcium variations using Fluo-4, excited at 488 nm, 200 ms exposure). They are overlaid for presentation purposes. The period between a pair of images was 1.08 s. Scale bar is 5 µm.

REFERENCES AND NOTES

1. X. Lou, Sensing exocytosis and triggering endocytosis at synapses: Synaptic vesicle exocytosis-endocytosis coupling. *Front. Cell. Neurosci.* **12**, 66 (2018).

2. T. Maritzen, V. Haucke, Coupling of exocytosis and endocytosis at the presynaptic active zone. *Neurosci. Res.* **127**, 45–52 (2018).

3. S. Watanabe, B. R. Rost, M. Camacho-Pérez, M. W. Davis, B. Söhl-Kielczynski, C. Rosenmund, E. M. Jorgensen, Ultrafast endocytosis at mouse hippocampal synapses. *Nature* **504**, 242–247 (2013).

4. R. Heidelberger, Z. Y. Zhou, G. Matthews, Multiple components of membrane retrieval in synaptic terminals revealed by changes in hydrostatic pressure. *J. Neurophysiol.* **88**, 2509–2517 (2002).

5. U. Djakbarova, Y. Madraki, E. T. Chan, C. Kural, Dynamic interplay between cell membrane tension and clathrin-mediated endocytosis. *Biol. Cell.* **113**, 344–373 (2021).

6. X. S. Wu, S. Elias, H. Liu, J. Heureaux, P. J. Wen, A. P. Liu, M. M. Kozlov, L. G. Wu, Membrane tension inhibits rapid and slow endocytosis in secretory cells. *Biophys. J.* **113**, 2406–2414 (2017).

7. Z. Shi, Z. T. Graber, T. Baumgart, H. A. Stone, A. E. Cohen, Cell membranes resist flow. *Cell* **175**, 1769–1779.e13 (2018).

8. J. E. Heuser, T. S. Reese, Evidence for recycling of synaptic vesicle membrane during transmitter release at the frog neuromuscular junction. *J. Cell Biol.* **57**, 315–344 (1973).

9. D. Zenisek, Vesicle association and exocytosis at ribbon and extraribbon sites in retinal bipolar cell presynaptic terminals. *Proc. Natl. Acad. Sci. U.S.A.* **105**, 4922–4927 (2008).

10. T. Vaithianathan, D. Henry, W. Akmentin, G. Matthews, Nanoscale dynamics of synaptic vesicle trafficking and fusion at the presynaptic active zone. *eLife* **5**, e13245 (2016).

11. L. Lagnado, A. Gomis, C. Job, Continuous vesicle cycling in the synaptic terminal of retinal bipolar cells. *Neuron* **17**, 957–967 (1996).

12. H. von Gersdorff, E. Vardi, G. Matthews, P. Sterling, Evidence that vesicles on the synaptic ribbon of retinal bipolar neurons can be rapidly released. *Neuron* **16**, 1221–1227 (1996).

13. S. Mennerick, G. Matthews, Ultrafast exocytosis elicited by calcium current in synaptic terminals of retinal bipolar neurons. *Neuron* **17**, 1241–1249 (1996).

14. H. von Gersdorff, G. Matthews, Dynamics of synaptic vesicle fusion and membrane retrieval in synaptic terminals. *Nature* **367**, 735–739 (1994).

15. G. Neves, L. Lagnado, The kinetics of exocytosis and endocytosis in the synaptic terminal of goldfish retinal bipolar cells. *J. Physiol.* **515** (Pt 1), 181–202 (1999).

16. J. Burrone, L. Lagnado, Synaptic depression and the kinetics of exocytosis in retinal bipolar cells. *J. Neurosci.* **20**, 568–578 (2000).

17. D. Zenisek, J. A. Steyer, M. E. Feldman, W. Almers, A membrane marker leaves synaptic vesicles in milliseconds after exocytosis in retinal bipolar cells. *Neuron* **35**, 1085–1097 (2002).

18. A. Llobet, V. Beaumont, L. Lagnado, Real-time measurement of exocytosis and endocytosis using interference of light. *Neuron* **40**, 1075–1086 (2003).

19. W. Shin, L. Ge, G. Arpino, S. A. Villarreal, E. Hamid, H. Liu, W.-D. Zhao, P. J. Wen, H.-C. Chiang, L.-G. Wu, Visualization of membrane pore in live cells reveals a dynamic-pore theory governing fusion and endocytosis. *Cell* **173**, 934–945.e12 (2018).

20. J. W. Taraska, D. Perrais, M. Ohara-Imaizumi, S. Nagamatsu, W. Almers, Secretory granules are recaptured largely intact after stimulated exocytosis in cultured endocrine cells. *Proc. Natl. Acad. Sci. U.S.A.* **100**, 2070–2075 (2003).

21. P. Sens, J. Plastino, Membrane tension and cytoskeleton organization in cell motility. *J. Phys. Condens. Matter* **27**, 273103 (2015).

22. D. Zenisek, G. Matthews, Calcium action potentials in retinal bipolar neurons. *Vis. Neurosci.* **15**, 69–75 (1998).

23. S. J. Bussell, D. L. Koch, D. A. Hammer, Effect of hydrodynamic interactions on the diffusion of integral membrane proteins: Diffusion in plasma membranes. *Biophys. J.* **68**, 1836–1849 (1995).

24. A. D. Dupuy, D. M. Engelman, Protein area occupancy at the center of the red blood cell membrane. *Proc. Natl. Acad. Sci. U.S.A.* **105**, 2848–2852 (2008).

25. O. M. Zakharova, A. A. Rosenkranz, A. S. Sobolev, Modification of fluid lipid and mobile protein fractions of reticulocyte plasma membranes affects agonist-stimulated adenylate cyclase. Application of the percolation theory. *Biochim. Biophys. Acta* **1236**, 177–184 (1995).

26. F. Brochard-Wyart, N. Borghi, D. Cuvelier, P. Nassoy, Hydrodynamic narrowing of tubes extruded from cells. *Proc. Natl. Acad. Sci. U.S.A.* **103**, 7660–7663 (2006).

27. A. Datar, T. Bornschlogl, P. Bassereau, J. Prost, P. A. Pullarkat, Dynamics of membrane tethers reveal novel aspects of cytoskeleton-membrane interactions in axons. *Biophys. J.* **108**, 489–497 (2015).

28. W. M. Morton, K. R. Ayscough, P. J. McLaughlin, Latrunculin alters the actin-monomer subunit interface to prevent polymerization. *Nat. Cell Biol.* **2**, 376–378 (2000).

29. M. Graffe, D. Zenisek, J. W. Taraska, A marginal band of microtubules transports and organizes mitochondria in retinal bipolar synaptic terminals. *J. Gen. Physiol.* **146**, 109–117 (2015).

30. C. Job, L. Lagnado, Calcium and protein kinase C regulate the actin cytoskeleton in the synaptic terminal of retinal bipolar cells. *J. Cell Biol.* **143**, 1661–1672 (1998).

31. H. von Gersdorff, G. Matthews, Inhibition of endocytosis by elevated internal calcium in a synaptic terminal. *Nature* **370**, 652–655 (1994).

32. R. Heidelberger, C. Heinemann, E. Neher, G. Matthews, Calcium dependence of the rate of exocytosis in a synaptic terminal. *Nature* **371**, 513–515 (1994).

33. M. Midorikawa, M. Tachibana, Fusion sites of synaptic vesicles in goldfish retinal bipolar cells. *Neurosci. Res.* **55**, S66 (2006).

34. R. Heidelberger, G. Matthews, Calcium influx and calcium current in single synaptic terminals of goldfish retinal bipolar neurons. *J. Physiol.* **447**, 235–256 (1992).

35. M. Morad, Y. E. Goldman, D. R. Trentham, Rapid photochemical inactivation of Ca^{2+}–antagonists shows that Ca^{2+} entry directly activates contraction in frog heart. *Nature* **304**, 635–638 (1983).

36. D. Feldmeyer, P. Zollner, B. Pohl, W. Melzer, Calcium current reactivation after flash-photolysis of nifedipine in skeletal muscle fibers of the frog. *J. Physiol. Lond.* **487**, 51–56 (1995).

37. A. Quan, A. B. McGeachie, D. J. Keating, E. M. van Dam, J. Rusak, N. Chau, C. S. Malladi, C. Chen, A. McCluskey, M. A. Cousin, P. J. Robinson, Myristyl trimethyl ammonium bromide and octadecyl trimethyl ammonium bromide are surface-active small molecule dynamin inhibitors that block endocytosis mediated by dynamin I or dynamin II. *Mol. Pharmacol.* **72**, 1425–1439 (2007).

38. P. Linares-Clemente, J. L. Rozas, J. Mircheski, P. García-Junco-Clemente, J. A. Martínez-López, J. L. Nieto-González, M. E. Vázquez, C. O. Pintado, R. Fernández-Chacón, Different dynamin blockers interfere with distinct phases of synaptic endocytosis during stimulation in motoneurones. *J. Physiol. Lond.* **593**, 2867–2888 (2015).

39. H. C. Chiang, W. Shin, W. D. Zhao, E. Hamid, J. Sheng, M. Baydyuk, P. J. Wen, A. Jin, F. Momboisse, L. G. Wu, Post-fusion structural changes and their roles in exocytosis and endocytosis of dense-core vesicles. *Nat. Commun.* **5**, 3356 (2014).

40. N. C. Gauthier, T. A. Masters, M. P. Sheetz, Mechanical feedback between membrane tension and dynamics. *Trends Cell Biol.* **22**, 527–535 (2012).

41. A. Paraschiv, T. J. Lagny, C.V. Campos, E. Coudrier, P. Bassereau, A. Šarić, Influence of membrane-cortex linkers on the extrusion of membrane tubes. *Biophys. J.* **120**, 598–606 (2021).

42. A. E. Cohen, Z. Shi, Do cell membranes flow like honey or jiggle like jello? *Bioessays* **42**, e1900142 (2020).

43. A. Kusumi, K. G. Suzuki, R. S. Kasai, K. Ritchie, T. K. Fujiwara, Hierarchical mesoscale domain organization of the plasma membrane. *Trends Biochem. Sci.* **36**, 604–615 (2011).

44. N. Borghi, F. Brochard-Wyart, Tether extrusion from red blood cells: Integral proteins unbinding from cytoskeleton. *Biophys. J.* **93**, 1369–1379 (2007).

45. P. Li, A. T. Bademosi, J. Luo, F. A. Meunier, Actin remodeling in regulated exocytosis: Toward a mesoscopic view. *Trends Cell Biol.* **28**, 685–697 (2018).

46. C. Joselevitch, D. Zenisek, Imaging exocytosis in retinal bipolar cells with TIRF microscopy. *J. Vis. Exp.* **2009**, e1305 (2009).

47. N. Dominguez, M. Rodriguez, J. D. Machado, R. Borges, Preparation and culture of adrenal chromaffin cells. *Methods Mol. Biol.* **846**, 223–234 (2012).

48. A. D. Edelstein, M. A. Tsuchida, N. Amodaj, H. Pinkard, R. D. Vale, N. Stuurman, Advanced methods of microscope control using μManager software. *J. Biol. Methods* **1**, e10 (2014).

49. U. Bockelmann, P. Thomen, B. Essevaz-Roulet, V. Viasnoff, F. Heslot, Unzipping DNA with optical tweezers: High sequence sensitivity and force flips. *Biophys. J.* **82**, 1537–1553 (2002).

50. F. M. Hochmuth, J. Y. Shao, J. Dai, M. P. Sheetz, Deformation and flow of membrane into tethers extracted from neuronal growth cones. *Biophys. J.* **70**, 358–369 (1996).

51. E. A. Evans, Bending elastic modulus of red blood cell membrane derived from buckling instability in micropipet aspiration tests. *Biophys. J.* **43**, 27–30 (1983).

52. J. Coelho Neto, U. Agero, R. T. Gazzinelli, O. N. Mesquita, Measuring optical and mechanical properties of a living cell with defocusing microscopy. *Biophys. J.* **91**, 1108–1115 (2006).

53. T. Betz, M. Lenz, J. F. Joanny, C. Sykes, ATP-dependent mechanics of red blood cells. *Proc. Natl. Acad. Sci. U.S.A.* **106**, 15320–15325 (2009).

54. H. Kanda, J. G. Gu, Membrane mechanics of primary afferent neurons in the dorsal root ganglia of rats. *Biophys. J.* **112**, 1654–1662 (2017).

55. D. G. Lowe, Distinctive image features from scale-invariant keypoints. *Int. J. Comput. Vis.* **60**, 91–110 (2004).

56. M. B. Smith, H. Li, T. Shen, X. Huang, E. Yusuf, D. Vavylonis, Segmentation and tracking of cytoskeletal filaments using open active contours. *Cytoskeleton* **67**, 693–705 (2010).

57. M. Kang, C. A. Day, A. K. Kenworthy, E. DiBenedetto, Simplified equation to extract diffusion coefficients from confocal FRAP data. *Traffic* **13**, 1589–1600 (2012).

58. P. G. Saffman, M. Delbruck, Brownian motion in biological membranes. *Proc. Natl. Acad. Sci. U.S.A.* **72**, 3111–3113 (1975).

59. S. J. Bussell, D. L. Koch, D. A. Hammer, Effect of hydrodynamic interactions on the diffusion of integral membrane proteins: Tracer diffusion in organelle and reconstituted membranes. *Biophys. J.* **68**, 1828–1835 (1995).

60. D. Needham, R. M. Hochmuth, A sensitive measure of surface stress in the resting neutrophil. *Biophys. J.* **61**, 1664–1670 (1992).

61. J. Dai, M. P. Sheetz, Axon membrane flows from the growth cone to the cell body. *Cell* **83**, 693–701 (1995).

62. P. Sens, Rigidity sensing by stochastic sliding friction. *Europhys. Lett.* **104**, 38003 (2013).

63. G. T. Charras, J. C. Yarrow, M. A. Horton, L. Mahadevan, T. J. Mitchison, Non-equilibration of hydrostatic pressure in blebbing cells. *Nature* **435**, 365–369 (2005).

64. E. Moeendarbary, L. Valon, M. Fritzsche, A. R. Harris, D. A. Moulding, A. J. Thrasher, E. Stride, L. Mahadevan, G. T. Charras, The cytoplasm of living cells behaves as a poroelastic material. *Nat. Mater.* **12**, 253–261 (2013).
